# Supplementary material for: Vittrup Man–The life-history of a genetic foreigner in Neolithic Denmark
Source: PLoS One. 2024 Feb 14;19(2):e0297032. doi: 10.1371/journal.pone.0297032 (PMC10866469; doi:10.1371/journal.pone.0297032)
Supplement: S1 File — (PDF) [file pone.0297032.s001.pdf]

Supporting Information

SI.1: The archaeology of Vittrup Bog

Anders Fischer, Per Lysdahl and Karl-Göran Sjögren

Find circumstances

The skeletal remains of Vittrup Man were found in a low-lying meadow near the village of Vittrup in Vendsyssel, NW-Denmark (Fig SI.1.1). They came to the light of day in 1915 close to the bottom of an about 3½ metre deep peat cut (Fig SI.1.2). The find report does not indicate that remains of a complete skeleton were present in the pit. Potentially, parts of the corpse may, however, for natural reasons have flowed away after the deposition, as is evidenced for other bog skeletons c.f. [1].

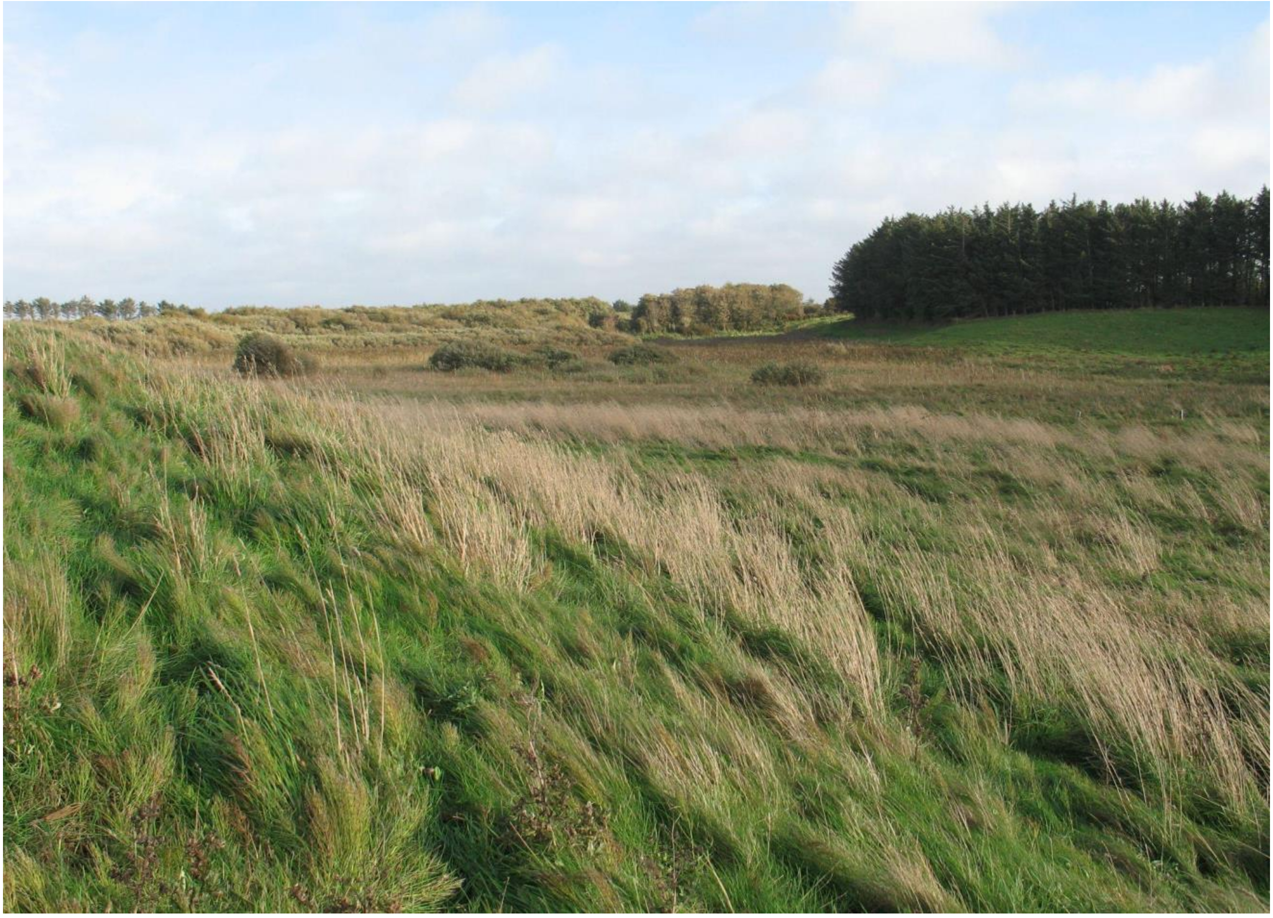

Fig SI.1.1. Everything now breathes peace at the site in the river valley, where the dramatically maltreated remains of Vittrup Man were deposited in Neolithic time. The peat cut from 1915 has long since been filled in and overgrown. Photo, seen towards the southeast, Anders Fischer 2018.

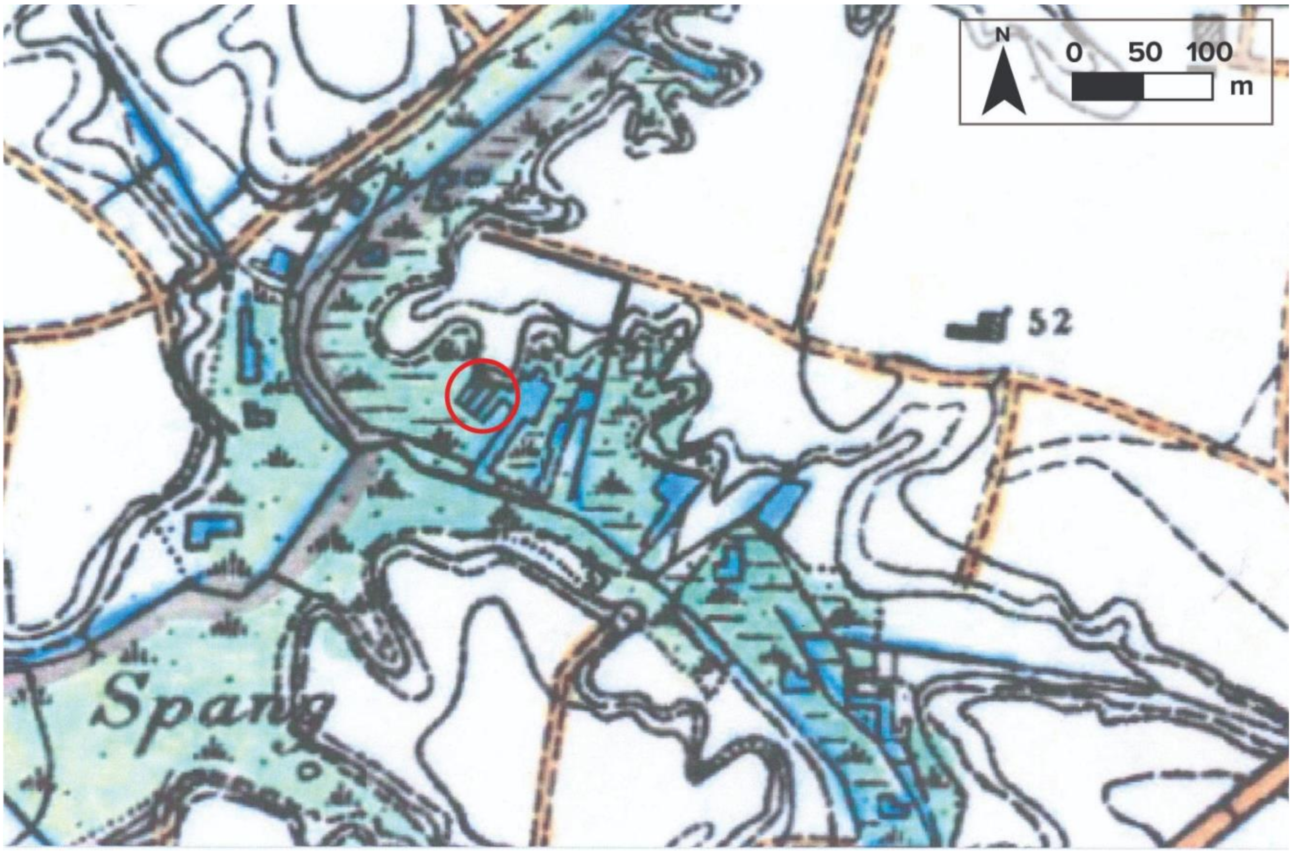

Fig SI.1.2. The approximate find spot for Vittrup Man (red circle), located close to the relatively steep side of a small river valley. Here the terrain rises 6 metres over a short distance. The equidistance of the height curves of the map is 5 Danish feet (1.57 m). Blue polygons symbolise peat cuts. The map is produced via the database 'Fund og Fortidsminder', curated by the Danish Agency for Culture and Palaces. Map: © Agency for Data Supply and Infrastructure. The map has the designation L32 and was originally measured in 1885.

It was only when the peat diggers encountered a ceramic vessel near the human bones that they found the situation so interesting that bids were sent for the local museum. The head of Vendsyssel Historical Museum, Valdemar Friis, showed up the next day and arranged a brief archaeological investigation in collaboration with the peat digging crew. He noted that the cut was so large that five men could sit next to each other and peel finds out of the peat wall. The bottom of the pot was still *in situ* but had gone in several pieces. It was located either lowermost in a branchy peat, or uppermost in the sediment below, which was referred to as "solid clay" (i.e. a marine deposit of Holocene or Late Glacial age). By searching the wet soil with their fingers, they managed to find a number of additional shards. In the peat wall half a metre away and perhaps at about the same depth they encountered a wooden club. Alternatively, it was located 30–40 cm above the bottom of the peat, as one of the participants (the landowner's son) stated 26 years later. Anyhow, the digging team noticed a number of small pieces of wood, which were affected by fire, around the pot and the club. A few decimetres higher in the bog sediments, they encountered a wooden pole, which was cut pointed at one end.

A piece of shaped wood with a flat top appeared close to the ceramic vessel. This - about 30 cm wide, c. 5 cm thick and just over a metre long specimen - was interpreted as possibly a fragment of a log boat. 26 years later, the local landowner – son of the owner in 1915 and one of the peat diggers at that time - reported that next to the pot there was also a flat, square stone measuring approx. 40 cm horizontally and 20 cm vertically. He conjectured the pot had originally stood on top of this stone.

In addition to the human remains, three bones of Bovines (*Bos*) were brought home from the peat cut (SI.5). Valdemar Friis immediately reported his find to the main archaeological authority in Denmark, the National Museum in Copenhagen. It was only the ceramic vessel and the wooden club that had a closer interest to this museum's director, Sophus Müller, when he visited Vendsyssel Historical Museum some months later to inspect the year's harvest of archaeological finds. In return for a modest state subsidy, provincial museums at the time had to let the National Museum take over what it wanted from the locally collected prehistoric finds. This honourable fate befell the pot and the club. Müller stated on the occasion that these two objects had great scientific value and that they would be more easily accessible to scientists from home and abroad at the country's main museum. He added that the club was probably the only wooden weapon from the Stone Age known from Danish territory (according to a note in the local newspaper, Vendsyssel Tidende 24 July 1915).

The pot and the club are still in the National Museum (inventory numbers A28426-27). They have since been mentioned several times in literature, where they are presented as examples of Neolithic sacrificial activity in wetlands [2,153,3,52,4,5]. With the carbon-14 measurements now available (main text, Table 1), this date and interpretation can be extended to the human and the bovine bones.

Topography

The Vittrup site is located in a 6–8 m deep river valley with a flat bottom, in a place where it forms a T-junction (Figs SI.1.1-3). From here is just over 3 km as the crow flies to the southwest to the confluence of the stream with the contemporary coast. North of the present-day estuary, a 15 km long cliff, which is constantly being eroded by the sea, illustrates that the country stretched further west in Vittrup Man's days.

The landscape around the deposition site of Vittrup Man is characterised by large stretches of slightly undulating land, cut up by river valleys. The nearly level land surface represents a seabed formed in Late Glacial time more than 11,000 cal years BC [6,7]. A local uplift of the earth’s crust, following the retreat of the ice sheets of the last Ice Age, caused the area to emerge above the sea, and from then onwards watercourse erosion began to furrow the landscape. Continued climatic amelioration caused a rise of waters, and by around 6000 cal years BC the sea flooded the river valley and deposited marine layers up past ocean the

site [6,8–11]. When a relative land rise from round 4000 cal years BC onwards caused the marine water to disappear (cf. [12,13]) peat formation on the valley floor began, over the following millennia resulting in the several metres deep formation of sediment, the extraction of which gave rise to finding the ancient bones and artefacts presented here.

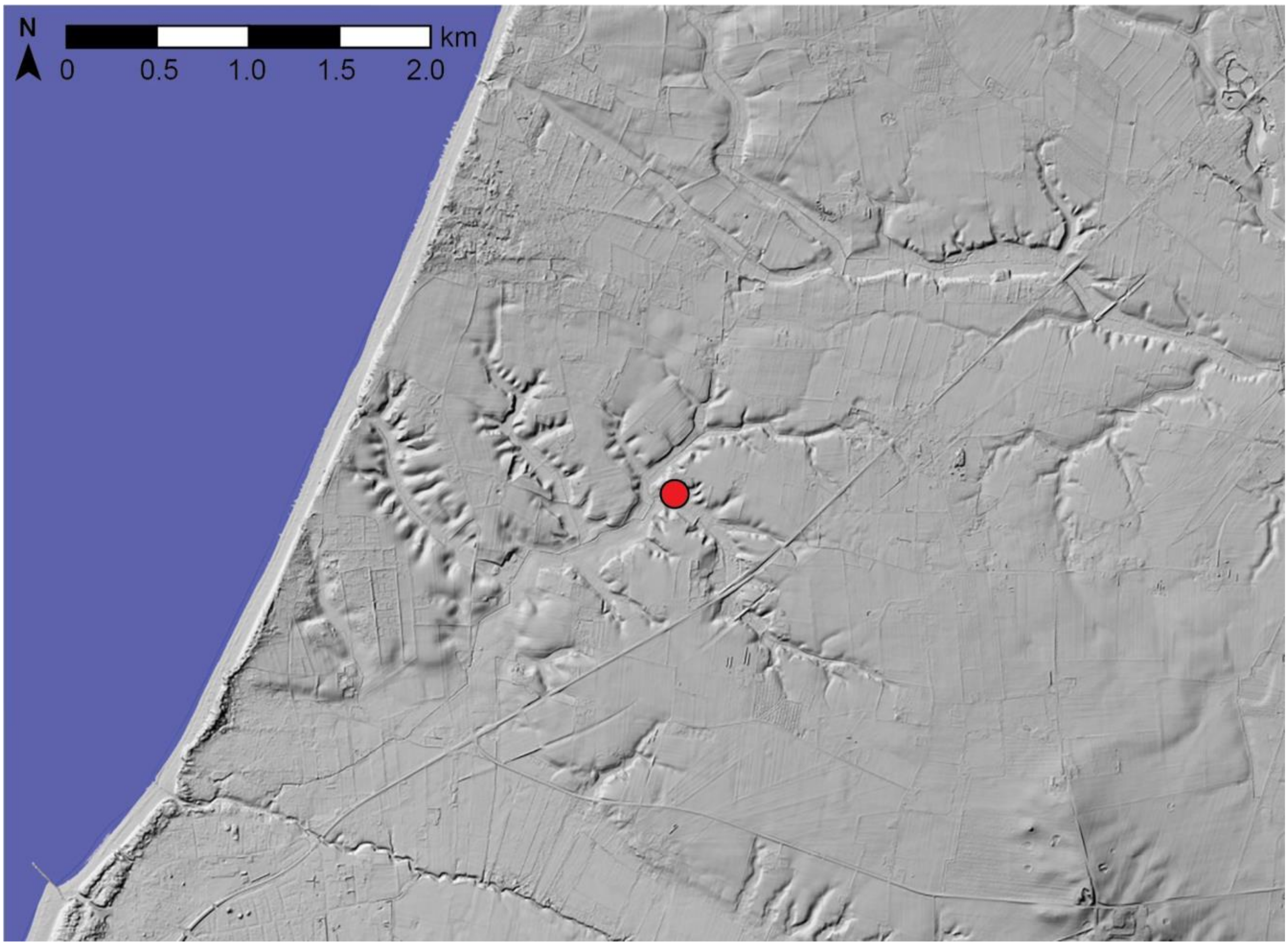

Fig SI.1.3. Today's relationship between land (grey) and sea (blue) around the Vittrup site (red dot). The map has been prepared using the database 'Fund & Fortidsminder' curated by the Danish Agency for Culture and Palaces. Terrain model 2015: © Hexagon.

Currently the sandy and silty soil with 4-6.9% clay ranks as the second poorest of North Jutland's five categories of agricultural land (Nordjyllands Amt 1975, map no. 11). Judging by the archaeological finds -conditions in other parts of Denmark, such easily cultivable soil was more valued among farmers in Vittrup Man's time [14–17]. Stray-found flint items from fields next to the site indicate that the local landscape was actually made use of during the Neolithic. A polished flint axe of thin-butted type demonstrates human activity in the area during the part of the Funnel Beaker Culture (FBC) epoch to which Vittrup Man belongs (Fund & Fortidsminder 10.01.02-9; VHM item no. 1967/273). Moreover,

from the location of megalithic monuments, recorded in the national archaeology database, Fund & Fortidsminder, it is evidenced that his deposition site was part of a landscape inhabited by people associated with the FBC. Within a 25 km half-circle 29 such monuments are known. In most cases these elite burials were demolished before it was determined if they represent dolmens or passage graves [18]. No-matter-what, they were important landmarks in the life of farmers of the same general date as Vittrup Man [19–21].

Other finds from the mire

The above-mentioned shards of a ceramic vessel could be put together and turned out to originate from a lugged vase. It has a round bottom and six vertical ears, placed slightly above its base (Fig SI.1.4). The vessel side is decorated in stab-and-drag (Danish: *furestik*) technique. Pottery with this shape and decoration is a characteristic of the Early Neolithic FBC's Volling style [22]. With its luxurious decoration it differs from most of the ceramic in the contemporary settlements. Pottery of this nature appears mainly in graves and in sacrificial contexts [23–26]. It belongs within an interval of time, which roughly spans 3800–3500 cal years BC [17,25,27,28], and consequently pre-dates Vittrup Man by several hundred years. This estimate of a chronological difference agrees with information from the excavation, indicating that the vessel was found a couple of decimetres deeper in the sediments than the human bones.

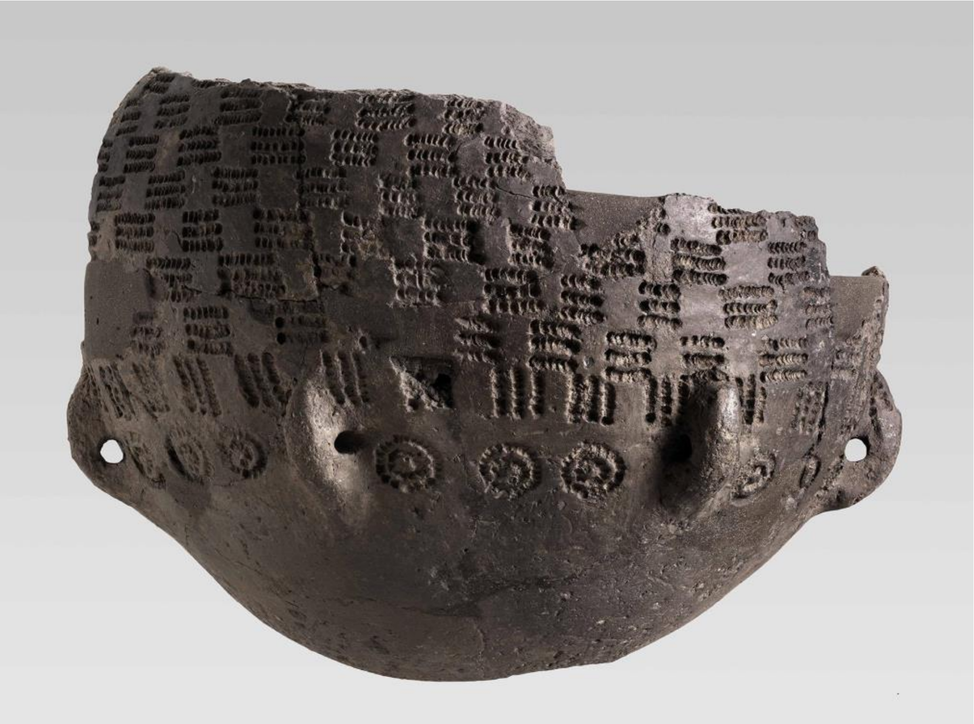

Fig SI.1.4. The refitted fragments of a lugged vase, which was probably deposited in the bog some centuries before the human remains. The rim part is missing. Maximum width 19 cm. Photo: John Lee, Danish National Museum.

The wooden club that was found close to Vittrup Man resembles a slightly oversized, modern baseball bat (Fig SI.1.5). In its current, preserved condition, it measures 28 cm in length and 5½–6½ cm in thickness. It is made of maple wood (*Acer campestre*), includes about 15 annual growth rings and has no traces of marrow nor the last growth season, implying it is produced from a relatively stout trunk [29]. The wood species is characterised by relatively large density and fracture strength [30–32]. Consequently, the item has been an effective weapon. It is difficult to achieve a reliable radiocarbon date from organic materials with preservatives, because of difficulties in removing all the artificially added substances [33]. Therefore, in the present study, no attempt at AMS dating the club has been made.

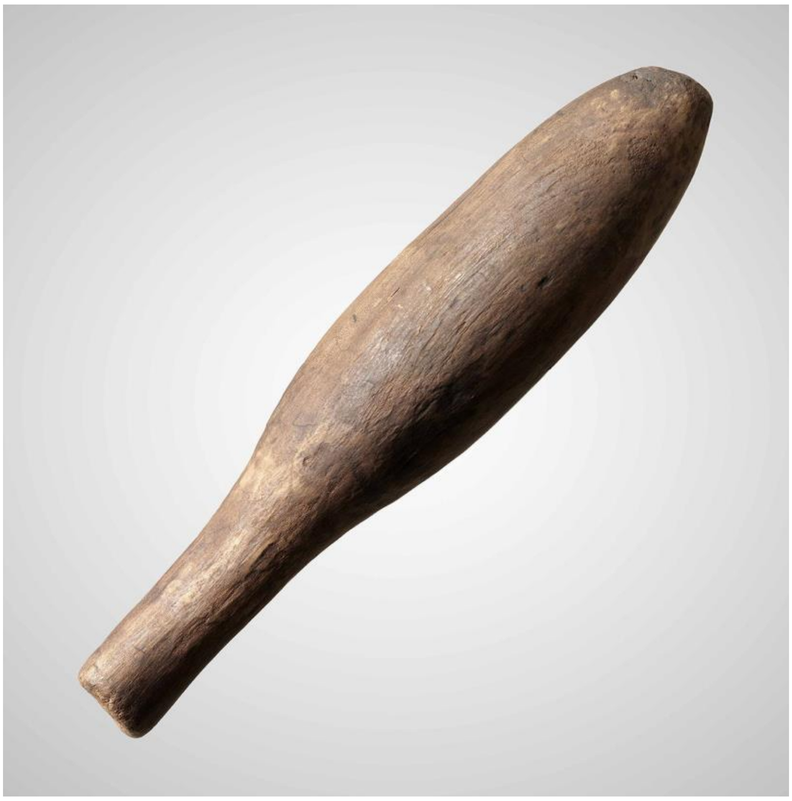

Fig SI.1.5. Murder weapon? Club of common maple. Its current length is 28 cm after some of the shaft broke off and disappeared during the exposure. Photo: John Lee, Danish National Museum.

Three bovine bones are available from the same small peat cut (SI.5). A metacarpal lay close to the human skeletal remains. Judged by its AMS date (main text, Table 1), it was deposited in the bog prior to Vittrup Man (Fig SI.1.6). The two other animal bones that were found in another part of the peat cut are from aurochs (*Bos primigenius*). Their ^{14}C dates indicate that one of them ended in the bog later than the human bones, while the other may be contemporary (Fig SI.1.6). Fauna-historically they confirm the current view that aurochs continued to be present locally long after the introduction of farming in Vendsyssel [34].

During previous peat excavation in the same bog, the skull of an aurochs had been found. The further fate of this find is not known, and its temporal relationship to Vittrup Man can therefore not be determined.

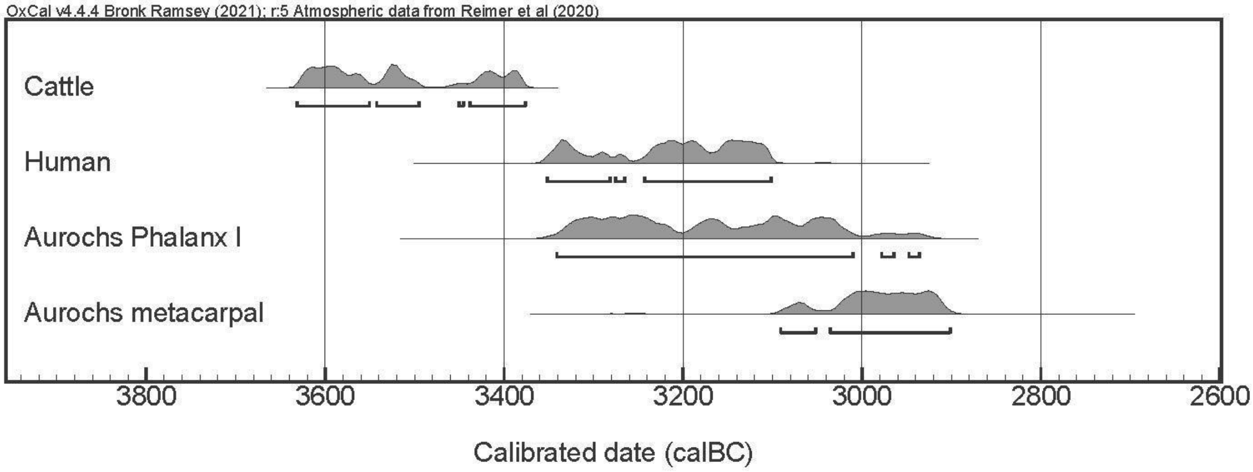

Fig SI.1.6. Probability graphs for radiocarbon dates of bones of domestic cattle, aurochs and homo, found in Vittrup Bog. The deposition of the three species probably represents different episodes. The date for the human is based on the combined date of four AMS measurements and an estimated marine reservoir offset of 33 years (see main text).

Depositional environment

The human and bovine bones as well as the wooden club were revealed in relatively fine states of preservation. For instance, collagen represents as much as 14% by weight of the skeletal remains (main text Table 1). Similarly good preservation is found in many other Danish human and bovine bog skeletons from the Neolithic period, whereas Stone Age skeletal material from kitchen middens and simple inhumation graves located in dry-land conditions usually have collagen values between 10 and 0 percent [35]. Mentioned categories of Danish archaeological localities all belong to moraine landscapes, the limestone-rich sediments of which are fundamental to their preservation of prehistoric skeletal material.

The fine preservation-condition of the organic remains from Vittrup indicates deposition in a water-saturated, oxygen-poor environment with rapid vegetation overgrowth and/or sediment cover. Such conditions can arise in small naturally formed lakes during periods of increasing humidity, and in wetlands influenced by beaver dam construction or sea level rise. They could also emerge in newly made peat cuts. Since the earliest traces currently known of peat extraction in Denmark date to the late Bronze Age [36], we favour the former alternatives of deposition in a naturally formed waterlogged setting.

SI.2: Vittrup Man's dentition - formation, wear and calculus

*Verner Alexandersen**

Vittrup Man's dentition plays a central role in the present study. Two of his teeth have provided us with samples for scientific analyses. These are an upper first premolar and a lower wisdom tooth. First of all, a sample for DNA analysis derives from the premolar. Moreover, we have used enamel from both of these teeth in the attempt to decide if he changed residence, and if so, at roughly what stage of life that happened. Additionally, we have used root increments of the mentioned wisdom tooth for the purpose of looking for signs of change in diet over time. Finally, we have sampled the dentition for calculus in an attempt at learning about what food species etc. this individual took in his mouth (SI.3).

On average the enamel of the tooth crowns of the upper first premolar (UP1) and lower third molar (LM3) of boys will form within the time intervals c. 2–7 years and 9–12 years after birth, respectively. Likewise, the beginning and end of root formation of males' lower wisdom teeth is in the range of c. 12–19 years based on the means and c. 14–20 years based on the medians, respectively (Table SI.2.1). Consequently, the values for Vittrup Man's tooth formations, stated in the main text, are approximations within uncertainty ranges generally exceeding one year.

Table SI.2.1. Tooth formation data in years for representative groups of males belonging to present-day populations of North America and Finland.

Stage of formation	Tooth	Range	Mean	1 St. dev.	Median	IQR
Crown initiation	UP1 (+4)	1.5-2.0	-	-	-	-
Crown completion		5.0-6.0	-	-	6.8	2.0
Root apex closure		12.0-13.0	-	-	14.0	4.0
Crown initiation	LM3 (-8)	-	9.2	1.0	9.8	6.2
Crown completion		-	12.0	1.2	13.7	5.0
Root apex closure		-	19.2	2.0	20.4	3.3

Data from [37–39]. IQR = interquartile ranges.

The wear in Vittrup Man's dentition is caused by mastication of hard and tough food and is concentrated to the largest cheek teeth, the molars. It resulted in slanting masticatory surfaces. The degree of slanting depends on the kind and consistency of the food and not least the biological age of the individual [40]. In several Danish Neolithic individuals, the slanting masticatory surfaces on the first and second molars are rather steep while the wear pattern in Late Mesolithic South Scandinavians is more horizontal on these molars [41].

The degree of wear on Vittrup Man's mandibular first molars is high, but the masticatory surfaces are still rather horizontal (Fig SI.2.1) as found in Late Mesolithic dentitions, not slanting as much as they often are in dentitions from the Funnel Beaker period. The well preserved mandibular second molar on the left side, however, shows the slanting masticatory surface characteristic for the wear pattern in mature Neolithic dentitions (Fig 4). The degree of wear on the second molar is two "wear degrees" [40] less than that of the first molars, which is typical for a dentition in the adult age group. In older age the first molars have the maximum degree of tooth wear and the second molars will eventually catch up with them. In summary: the wear pattern and the degrees of wear of the cheek teeth of Vittrup Man show a mixture of traits, typical for Danish skeletons of Late Mesolithic and Neolithic (Funnel Beaker) date, respectively.

Like many other individuals from the Funnel Beaker Culture epoch in Denmark, Vittrup Man grew calculus, which is mineralized plaque. As is typical for this phenomenon, his cheek teeth show the most abundant calculus formation (Fig SI.2.1). Its accumulation may have begun, although to a slight degree, soon after the eruption of the permanent teeth.

Calculus is usually divided into two categories: subgingival on the roots, supragingival on the enamel of the crown surfaces (Fig SI.10.1). Both kinds are documented in the photogrammetric model of a lower third molar (Fig SI.4.1), where a strip of such deposit is seen on the tooth crown and many small lumps adhere to the upper part of the root in what was formerly the gingival pocket with inflammatory gingiva. Both categories can result in severe periodontal disease (*paradentosis*). One of the microorganisms listed as potentially represented in the calculus are in fact known to be involved in periodontal disease (*Porphyromonas gingivalis*, if combined with other species of microbes). Other of the potentially represented species are, however, common in healthy oral cavities[42]. In the case of Vittrup Man the involvement of alveolar bone in inflammatory processes was still localized to few locations in the dentition.

His calculus is of the supragingival type. Although the chewing of hard foods will have removed some plaque and calculus, there will have been a gradual accumulation. Consequently, the hardened plaque on his teeth (SI.3) will probably carry information on his diet from the time of eruption of the extant teeth - from around the age of 7 onwards - to the time of his death.

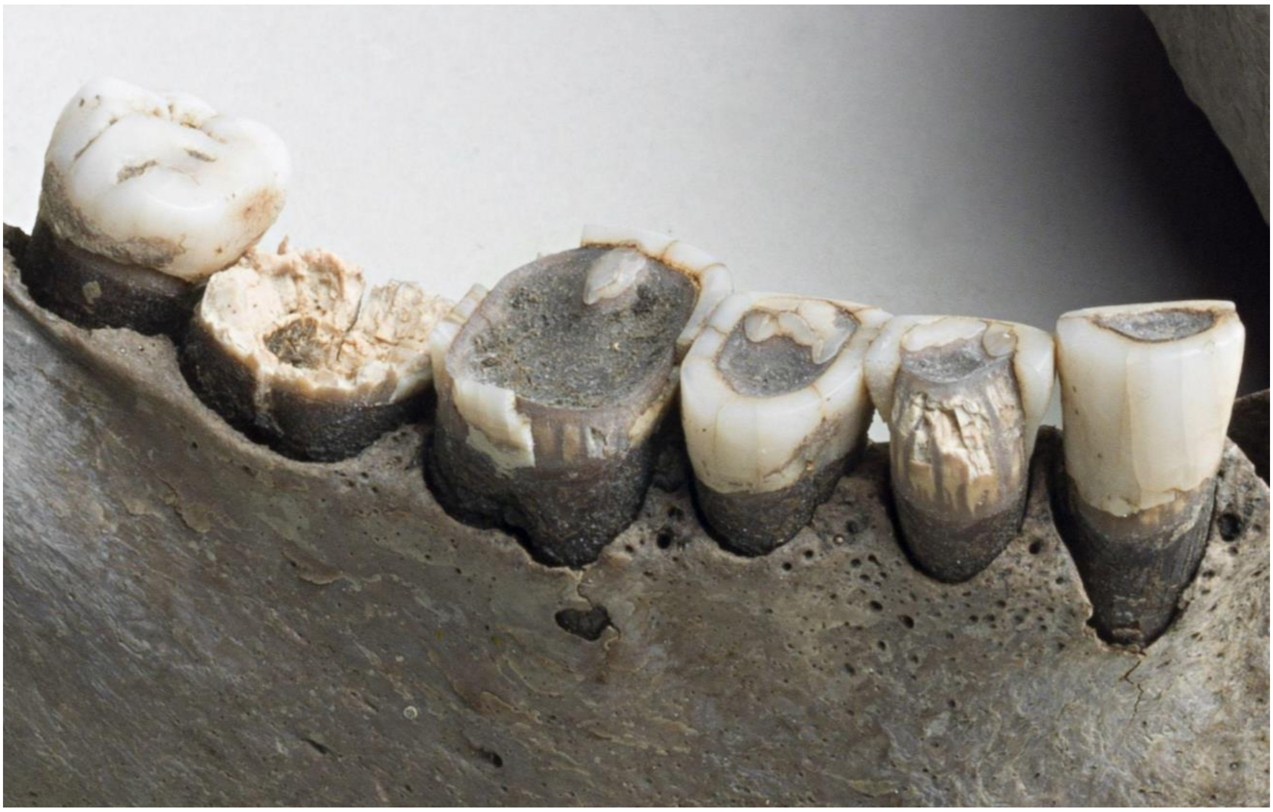

Fig SI.2.1. The right side of Vittrup Man's mandible with dental calculus on the enamel of the wisdom tooth and (partly preserved) on the second premolar. Porosity of the bone is observed next to the premolars (the smaller cheek teeth) and in between the teeth. Persisting dental plaque and calculus formation result in chronic inflammation of the gum that eventually leads to destruction of the alveolar bone. Here the bone porosity suggests beginning involvement of the alveolar bone. The wear of the first molar (third position from behind) is so pronounced that only a small 'island' of the thick layer of enamel on the chewing surface remains. Photo: Arnold Mikkelsen, Danish National Museum.

* Lecturer emeritus in dental anthropology in the Laboratory of Biological Anthropology, University of Copenhagen.

SI.3: Proteomic analysis of Vittrup Man's calculus

Gabriele Scorrano

Calculus was present on at least five of Vittrup Man's teeth (Figs SI.2.1 and SI.10.1). These were tooth -4, -7, 5-, 8- and -8 according to the nomenclature of Haderup (34, 37, 38, 45 and 48 according to the FDI two-digit system). Sampling was conducted on all of them and the material from four of them was merged and analysed. Protein extraction was performed on around 7 mg of dental calculus following the method proposed by Jersie-Christensen et al. [43]. The powder was demineralized overnight with 1 ml 15–20% acetic acid. Via centrifugation for 10 min at 2000 g the acetic acid was removed. Subsequently a lysis buffer (6M guanidine hydrochloride, 10mM chloroacetamide, 20mM Tris(2-carboxyethyl) phosphine Hydrochloride in 100mM TRIS pH 8.5) was added to the powder and the pH was adjusted to 7–9. The pellet was then crushed and incubated at 99 °C for 10 mins at 500 rpm. The sample was digested with rLysC (0.2 µg, Promega, Sweden) incubating under agitation at 37 °C for 2–4 hours. Subsequently, 25 mM Tris in 10% acetonitrile were used to dilute the sample to a final concentration of 0.6 M GuHCl. Another digestion was performed by trypsin (0.8 µg, Promega, Sweden) and incubation overnight at 37 °C under agitation. The digestion was stopped by acidifying the sample (pH <2) by 10% trifluoroacetic acid, then the proteins were collected in home-made C18 StageTips and stored in the freezer until mass spectrometry analysis.

The data analysis was performed on the sample and on the extraction, blank using MaxQuant version 1.6.2a [44] with oxidation (M), Acetyl (protein N-term), deamidation (NQ), and hydroxyproline set as a variable modifications and carbamidomethyl (C) as a fixed modification. Trypsin was set as a 'digestion enzyme' with a maximum of two missed cleavages allowed. For the identification, a minimum score of modified and unmodified peptides of 40 was used and a Peptide Spectral Match (PSM) and Protein false discovery rate (FDR) of 0.01 cutoff was set. All other parameters were left for the default for orbitrap mass spectrometers. Different searches by several databases were performed. Because dental calculus should be rich in oral microbiome and human proteins [45,46], a search against the Oral Signature Screening Databases (OSSD) was performed [46]. OSSD is a database with a restricted list of the most abundant oral microbes and human inflammatory response proteins commonly found in archaeological samples and common contaminants [46]. The OSSD run is useful to assess if the analysed samples contain ancient oral microbiome and human proteins. It cannot validate the results of the food-proteins; however, it is a screening method to evaluate the oral signature in archaeological dental calculus samples [46]. The dental calculus of the Vittrup sample shows a fair amount of human and oral microbiome proteins (Table SI.3.3; cf. SI.2) suggesting it would likely yield consumed food-proteins as well.

For the broad food-proteins screening, the entire SwissProt database (downloaded in January 2017) was used and then a specific database with all the species identified in the first run was built and then a search against

a FASTA file built using all the proteomes of the species identified by the first search following the workflow proposed by Scorrano and colleagues [47] was performed. A search against the FASTA file built using the proteome of *fabaceae* and *poaceae* was also performed and milk and dairy products consumption was eventually searched. The peptides obtained were then filtered to remove reverse and common contaminants and the protein group with a value of Razor + unique less than two were removed. For species validation, all the spectra identified were manually confirmed by BLAST search [48].

To avoid data misinterpretation, all the spectra associated have been manually validated, annotated, and inspected. Moreover, the rate of deamidation for asparagine and glutamine damage, consistently observed in ancient samples, was also evaluated to support the authenticity of the identified proteins by using the python tool proposed by Mackie et al. [49]. Vittrup Man shows an advanced rate of deamidation (Fig.SI.3.1), representing a preservation state compatible with ancient proteins [50,51].

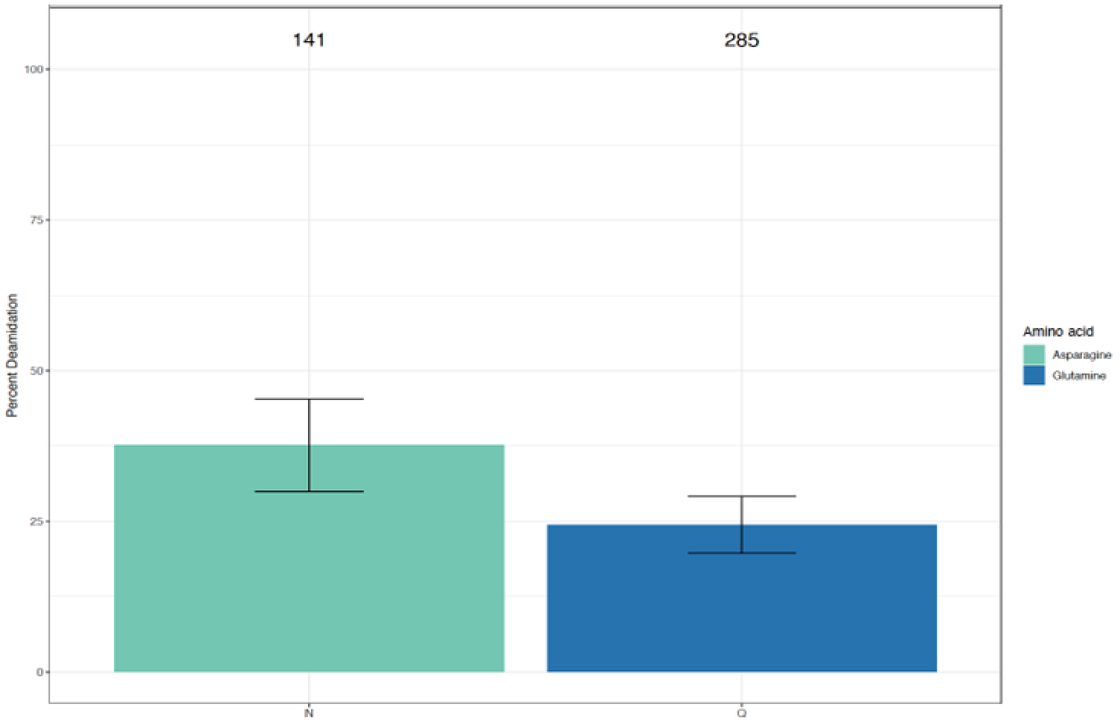

Fig SI.3.1. Overall percentage of deamidation for asparagine (N) and glutamine (Q) amino acids for the proteins found in the Vittrup dental calculus sample, graphically presented according to a standard within this field of research [49]. It shows the degradation of ancient proteins identified in the sample. The error bars represent standard deviation.

The outcome of the analysis in terms of food species demonstrated via the proteomic calculus analysis is summarised in Table SI.3.1. It reports the proteins identified, in italics the species identified and in bold the common names and the information coming from the MaxQuant run.

Table SI.3.1. Dietary proteins and species detected in the dental calculus sample by the sequences shown in Table SI.3.2.

Accession number	Protein name	Species	Razor + Unique peptides	Taxonomic diagnostic peptides	Unique + razor sequence coverage [%]	Number of each spectrum supported
XP_017920382.1, XP_017920383.1, XP_017920384.1, XP_027830506.1, KAG5203341.1	Collagen type I alpha 1 chain isoform X1, Collagen type I alpha 1 chain isoform X2, Collagen type I alpha 1 chain isoform X3; Collagen type I alpha 1 chain isoform X1, hypothetical protein JEQ12_002924	**ovine** (*Capra hircus* or *Ovis aries*)	5	1	6.8	486
XP_032284949.1	Collagen type I alpha 1 chain	**seal** (*Phoca vitulina*)	76	1	51	442
XP_035950567.1, XP_032284949.1	Collagen type I alpha 1 chain	**seal** (*Halichoerus grypus* or *Phoca vitulina*)	76	1	51	442
XP_030215358.1, XP_030215367.1	Collagen type I alpha 2 chain isoform X1, Collagen type I	**atlantic or baltic cod** (*Gadus morhua* or *Gadus*	2	1	1.4	13

	alpha 2 chain isoform X2	*morhua callarias*)				
XP_030196341.1, XP_030262871.1, XP_030262873.1	Collagen type I alpha 1 chain; Collagen type I alpha 1 chain isoform X1, Collagen type I alpha 1 chain isoform X3	**atlantic or baltic cod** (*Gadus morhua* or *Gadus morhua callarias*) or **gilt-head (sea) bream** (*Sparus aurata*)	3	1	4.7	5
XP_028020809.1, XP_036691628.1, XP_036691630.1, XP_036691631.1, XP_030695607.1, XP_033703059.1, XP_004282670.1	Collagen type I alpha 1 chain; Collagen type I alpha 1 chain isoform X1, Collagen type I alpha 1 chain isoform X2, Collagen type I alpha 1 chain isoform X3; Collagen type I alpha 1 chain; Collagen type I alpha 1 chain; Collagen type I alpha 1 chain	**Cetacean: whale** (*Balaenoptera acutorostrata scammoni* or *Balaenoptera musculus*) or **ocean dolphin** (*Globicephala melas* or *Tursiops truncatus* or *Orcinus orca*)	3	1	7.6	217

Razor + Unique peptides: number of peptides associated with each protein group identified; Taxonomic diagnostic peptides: number of species-specific peptides confirmed by BLAST search; Unique + razor sequence coverage (%): percentage of the sequence that is covered by the identified unique and razor peptides; Number of each spectrum supported: number of spectra which supported each protein group.

Table SI.3.2. Observed genus-diagnostic peptides in the dental calculus sample confirmed by BLAST search. The MaxQuant score referred to the best matched spectrum.

**Accession number Protein name**	**Sequence**	**Length**	**Mass**	**MaxQuant score**	**Number of each spectrum supported**
XP_017920382.1, XP_017920383.1, XP_017920384.1, XP_027830506.1, KAG5203341.1	GGPGSRGFPGSDGVAGPKG PAGER	24	2166.0566	78.202	1
XP_032284949.1	GFPGSDGVSGPKGPAGER	18	1670.8012	55.44	1
XP_035950567.1, XP_032284949.1	GDRGETGPSGPPGAPGAPG APGPVGPAGK	29	2464.2095	70.647	1
XP_030215358.1, XP_030215367.1	GAQGAPGATGFPGSSGR	17	1473.3961	108.32	1
XP_030196341.1, XP_030262871.1, XP_030262873.1	GPAGPPGATGFPGAAGR	17	1436.7161	255.52	1
XP_028020809.1, XP_036691628.1, XP_036691630.1, XP_036691631.1, XP_030695607.1, XP_033703059.1, XP_004282670.1	TGPPGPAGQDGRPGPPGPPG SRGQAGVMGFPGPK	34	3134.5468	136.02	1

Table SI.3.3. Proteins and species detected in the dental calculus sample by the search against the Oral Screening Databases. Razor + Unique peptides: number of peptides associated with each protein group identified; Unique peptides: number of species-specific peptides identified by the MaxQuant run; Unique + razor sequence coverage (%): percentage of the sequence that is covered by the identified unique and razor peptides; Number of each spectrum supported: number of spectrums which supported each protein group.

Accession Number	Protein name	Species	Razor + Unique peptides	Unique peptides	Unique + razor sequence coverage [%]	Number of each spectrum supported
P02452	Collagen type I alpha 1 chain	Homo sapiens	118	113	58.3	820
P08123	Collagen type I alpha 2 chain	Homo sapiens	94	94	65.2	509
P02461	Collagen type I alpha 3 chain	Homo sapiens	13	9	15.4	14
P02458	Collagen type 2 alpha 1 chain	Homo sapiens	7	6	6.3	33
P05997	Collagen type 5 alpha 2 chain	Homo sapiens	7	7	5.9	14
P20908	Collagen type 5 alpha 1 chain	Homo sapiens	4	4	2.6	4
P59665	Neutrophil defensin 1	Homo sapiens	4	4	20.2	7
P05109	Protein S100-A8	Homo sapiens	2	2	19.4	2
P68104	Elongation factor 1-alpha 1	Homo sapiens	2	2	4.1	3
F9PI58	Elongation factor Tu	Actinomyces sp. oral taxon 175 str. F0384	2	2	4.3	3
F9PJC7	L-lactate dehydrogenase	Actinomyces sp. oral taxon 175 str. F0384	2	2	6.0	3
FPK61, S3L148, C8NGI8, F9HFS5, Q7MT61, A8AUR7, Q8DVV3	Glyceraldehyde-3-phosphate dehydrogenase	Actinomyces sp. oral taxon 175 str. F0384 or Treponema maltophilum or Granulicatella adiacens or Streptococcus sp. oral taxon 056 str. F0418 or Porphyromonas gingivalis or Streptococcus gordonii or Streptococcus mutans serotype c	2	2	4.5	5

In summary, our dental calculus palaeoproteomics data provide an unprecedentedly detailed insight into the diet of a North European Stone Age individual, showing a diet that included animal protein from a variety of aquatic and terrestrial resources. Emphasis shall not be put on the lack of trace of plant food or milk/dairy products. The absence of specific food proteins does not necessarily mean that a particular food resource was not regularly ingested; it could also reflect a poor preservation environment in the oral cavity and/or the burial environment.

Moreover, our results show the benefit of using the proteomics approach in dental calculus analyses because it can identify species even when they are absent in the traditional archaeological records, where animal bones are typically few and fish remains most often totally absent.

The mass spectrometry proteomics data have been deposited to the ProteomeXchange Consortium via the PRIDE [52] partner repository with the dataset identifier PXD044743.

SI.4: Photogrammetric documentation prior to destructive sampling

Theis Zetner Trolle Jensen and Anders Fischer

Destructive sampling ethics

Prehistoric human skeletons of fine preservation quality are rarely encountered during archaeological excavations. The exceptionally well-preserved skeletal parts actually available are therefore attracting much attention these years, which has seen an increase in the invention and use of bio-archaeological methods for the study of human diet, genomes, etc. These molecular methods are based on destructive sampling. For instance, in the aim of genetic analysis, a whole tooth or petrous bone (*pars petrosa*) is often desired and will have to be expended to such a degree that future studies of its morphology, wear, etc. are largely inhibited and the exhibition quality of the skeleton in question may be compromised. This problem also applied in the case of the Neolithic skeletal remains of Vittrup Man, when our research strategy implied that an entire tooth would be used for analyses (SI.8 and SI.10). In this situation, we deemed it an ethical responsibility to at least document the tooth before carrying out destructive analysis. With the advent of 3D printing, it has become possible to print high-resolution models, which can act as placeholders for what was initially used for analyses. Anyhow, at a certain point in the analytical process we decided not to take any further destructive samples from this individual (cf. SI. 8). Relatively few teeth are available from Vittrup Man and we feel an obligation that as many as possible of these shall be at disposal for future generations of researchers. They will, no doubt, possess methods than can reveal scientifically crucial information which is currently totally out of reach.

In the case of Vittrup, the tooth selected for analysis was the left upper first premolar (UP1; +4). We performed photogrammetry of the specimen to create a high-resolution model in colour of the tooth, which can later be printed and inserted into the maxilla.

Methods

Photogrammetry was carried out as follows: The tooth was placed in tack on a small pedestal, on a turntable against a backdrop of contrasting colour and texture (light blue cloth). Photos were taken using focus stacking on an Olympus OMD-EM1 Mark II, where for each still eight photos are taken at different focal points and subsequently merged to obtain a high in-depth focal field. A total of 55 photographs were taken incrementally at 360° on two different arcs, for the distal end of the molar. Next, the same was done taking a subsequent 60 photos of the reverse proximal end, also at two different arcs.

Photo pre-processing for background masking and model creation and export was all done using Agistoft Metashape Professional (v. 1.6.5). After pre-processing, the photos of the proximal and distal end of the

molar were separately aligned and built into dense clouds. Next, the two chunks were aligned and merged into one. The combined model was then built into a meshed model, and finally into a textured model. Due to significant diffuse light reflectance from spots on the enamel, surface inconsistencies can be seen on the crown part of the replicate tooth. However, this will hardly be noticeable on a printed model.

After the creation of the textured model, the merged textured chunk was scaled according to the measurements obtained using a digital calliper. Lastly, the model was exported to the open wavefront format OBJ, to preserve readability. The OBJ and associated texture files are available here: https://doi.org/10.5281/zenodo.7802122 as external Supplementary.

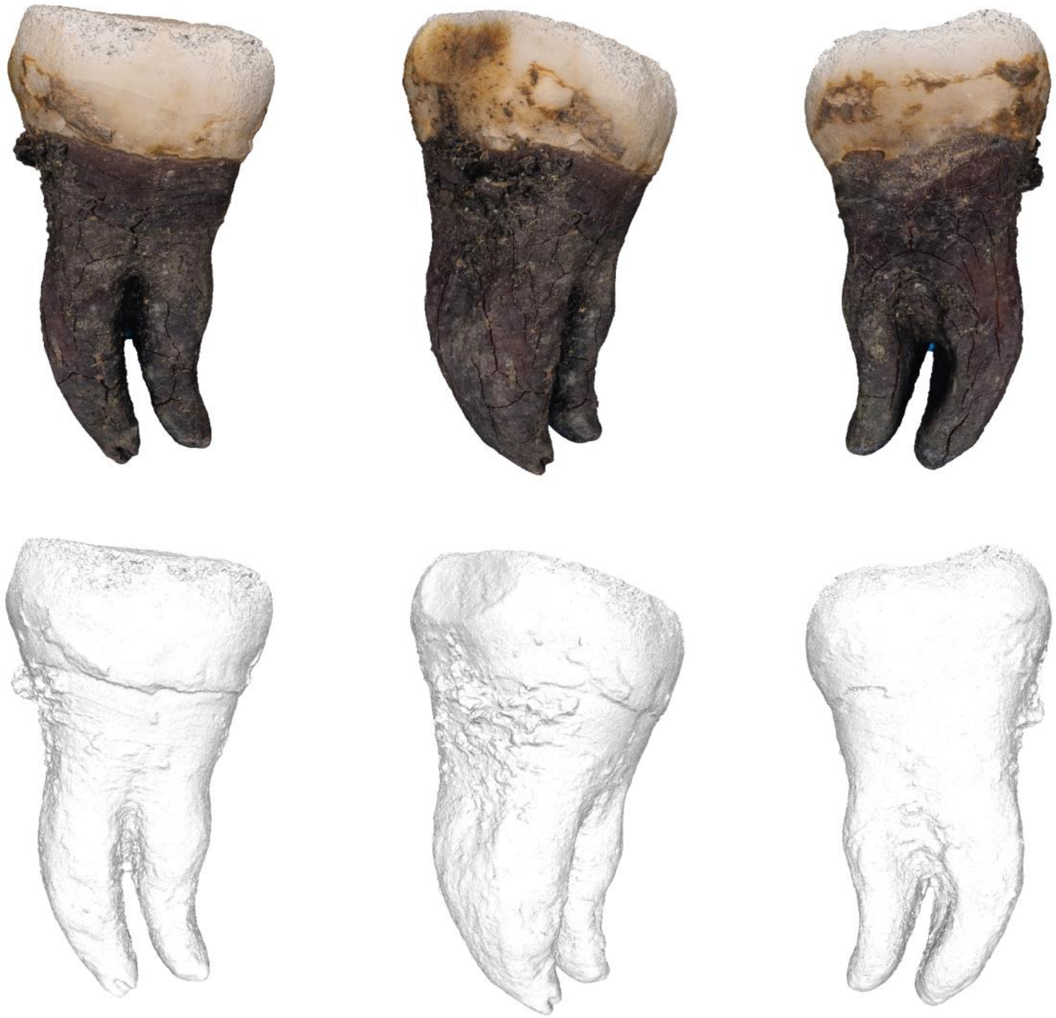

Fig SI.4.1. Photogrammetric model in textured and meshed view of Vittrup Man's tooth. Produced by T. Zetner Trolle Jensen.

SI.5: Aurochs and cattle bones from Vittrup Bog

Anne Birgitte Gotfredsen

In the stores of Vendsyssel Historiske Museum (file Nr. 54-1968) are kept three bones which at the time of discovery were inspected by Magnus Degerbøl at the Zoological Museum but otherwise left unnoticed until today. Thanks to AMS dating and the measuring of their strontium isotopic and dietary stable isotope (δ^{13}C, δ^{15}N and δ^{34}S; Tables 1, SI.8.1, SI.9.1 and SI.11.1) values they have been shown to be of importance to the understanding of Vittrup Man's remarkable life history some five thousand years ago. In addition, the combination of their radiocarbon dates (main text, Table 1) and their zoological species determination adds to the zoogeographic knowledge on late surviving aurochs (*Bos primigenius*) in Denmark.

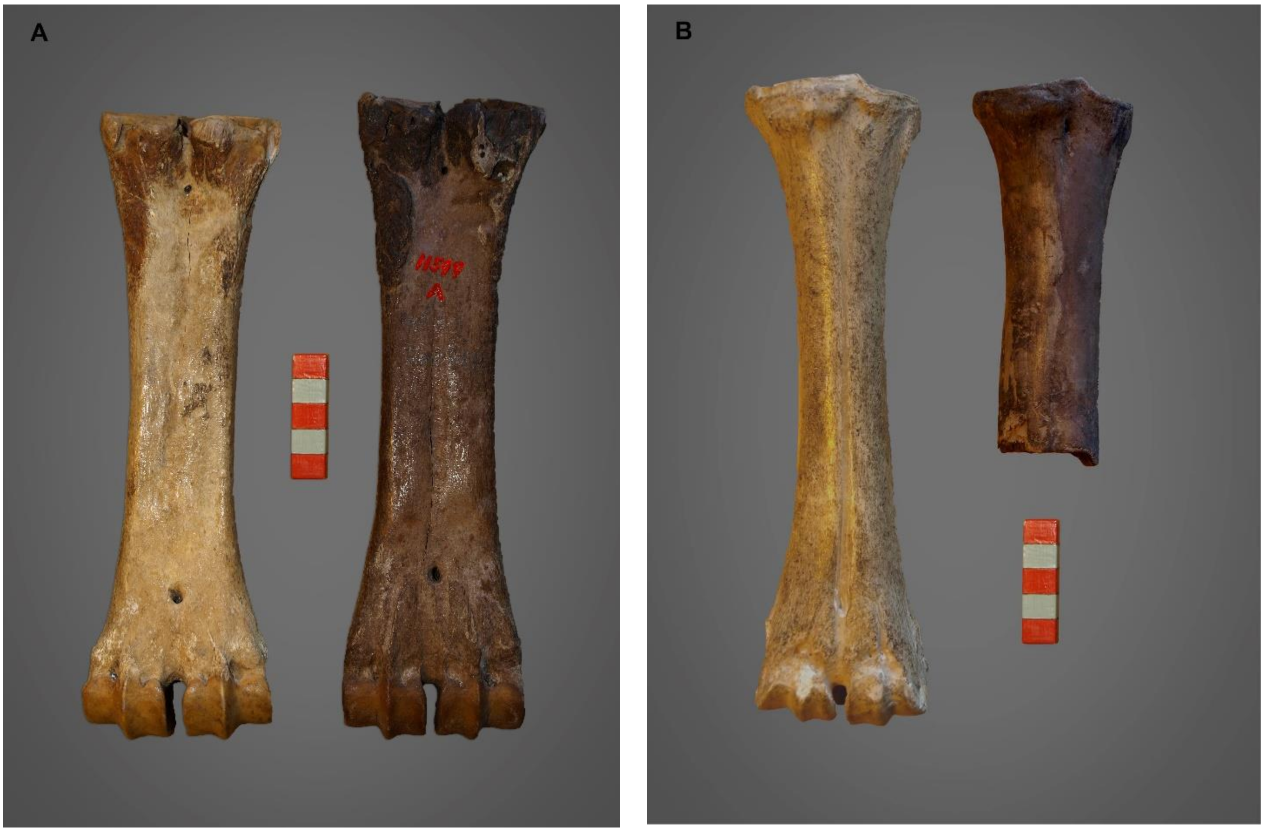

Fig SI.5.1. Bovid bones from Vittrup Bog: A) metacarpus *from female aurochs from Ullerslev (left) and the Vittrup specimen VHM 11599A (right); B)* metacarpus *from a modern jersey cow (left) and the VHM 11602A specimen (right). Scales in cm. Photos: A.B. Gotfredsen.*

VHM 11599A and VHM 11599B

VHM 11599A is a left metacarpal (*metacarpus*) of an aurochs (*Bos primigenius*) (Fig SI.5.1A). Its dimensions (Table SI.5.1) are within the range of Danish aurochs cows [53]: Table 11[53], and are larger

than those of the smallest female aurochs specimen known from Denmark (Ullerslev). In addition, its greatest length (GL), breadth of the proximal part (Bp) and smallest breadth of the diaphysis (SD), respectively, is larger than those of Neolithic domestic cattle bulls [53]: Table 11]. The metacarpal exhibits *exostosis* proximally on the caudal aspect and to a minor degree across the diaphysis.

VHM 11599B is a first toe bone of the forelimb, *phalanx proximalis*, anterior of an aurochs (*Bos primigenius*). This robust bone has weakly developed *exostosis* ventrally on the peripheral face.

Both bones are well preserved, exhibiting almost the same colour and patina. None of them shows cut marks or other signs of anthropogenic impact. The bones may well have been articulated and belonged to the same individual despite the coloration of the toe bone being a bit darker than the articular surface of the metacarpus. Based on their AMS dates (Table 1 and Figure SI.1.6), however, they are most likely the result of two independent depositional episodes. They both, in particular the metacarpal, exhibit *exostosis*, a phenomenon known to occur in older aurochs individuals [54]. *Exostosis* is also seen in conjunction with other features such as lipping of the articular surface, on cattle bones from individuals used as draft animals [54]. Such features, however, cannot be seen on VHM 11599A and 11599B. Therefore, the *exostosis* on these bones is interpreted as an indication of advanced age.

When comparing the measurements of the present investigation it seems it is the same bones that were listed by Degerbøl & Fredskild [53] under the site name of Vittrup.

VHM 11602A

Upper part of a left metacarpal, presumably from domestic cattle (*Bos taurus*) (Fig SI.5.1B; cf. SI 1). The bone's dimensions match those of an extant Jersey cow. Some reservation to the species identification must however be taken since the lower part of the bone is missing, making it impossible to establish whether the bone had a loose still not fused distal epiphysis and therefore could derive from a subadult not fully grown individual. The thickness of the bone wall and the beginning of exfoliation of the bone surfaces could indicate a subadult individual. The bone, however, originates from an almost full-grown individual and thus suggests that it is domestic cattle. Proximally in the medial articular surface, the natural indention exhibits a perforation. This perforation seems not to have been inflicted intentionally by a tool although it cannot be fully ruled out. There are no cut marks present on the bone. The few tiny scratch marks may have been caused by trampling. The bone is broken amidst the diaphysis. The breakage seems to be of an older age and the bone possibly was broken prior to deposition. It is, however, not typical for marrow-split bones and could be a dry breakage of the bone that had happened after deposition.

Tabel SI.5.1 Dimensions of aurochs and cattle bones in mm from Vittrup Bog.

**Element**	**side**	**GL**	**Bp**	**Dp**	**SD**	**Bd**	**Dd**
Metacarpus VHM 11599A	sin, intact	250.10	74.52	45.26	43.34	67.83	39.25
Metacarpus VHM 11602A	sin, proximal		55.21	33.17			
**Element**	**part**	**Glpe**	**Bp**	**SD**	**Bd**		
Phalanx 1 VHM 11599B	anterior	69.91	36.21	30.8	33.35		

Definitions of measurements according to von den Driesch (1976).

SI.6: The genomic ancestry of the Vittrup and Svinninge Vejle individuals

Tharsika Vimala

Material

The present study focuses on skeletal remains of two individuals, dating to the Neolithic age, found in the Danish sites of Vittrup and Svinninge Vejle. Their nuclear genetic characterisation was performed at the Centre for GeoGenetics at the University of Copenhagen. Results of this work have already been presented in a paper focused on the human genetics of Mesolithic and Neolithic Eurasia [55], and are now expanded upon in order to investigate the genetic ancestries of the Vittrup and Svinninge individuals in a new context relative to a set of genomic profiles for individuals associated with the Pitted Ware Culture (PWC) published by Coutinho et al., 2020 [56]. The genetic analysis was based on samples from a left upper first premolar (UP1; +4) and a left *pars petrosa*, respectively. Analysis of the post-mortem damage patterns carried out in Allentoft et al., [55] revealed fractions of C-to-T transitions at the 5'position on the sequenced DNA fragments of 23.1% and 33.3%, and mitochondrial contamination levels of 0.04–0.9% and 1.3–2.0% for Vittrup and Svinninge Vejle, respectively, confirming the presence of ancient DNA. We obtained a final coverage of 0.05X of Vittrup Man and 3.8X for Svinninge Vejle. Sex determination was carried out by calculating the ratio of reads aligning to either of the sex chromosomes, X or Y, and revealed that both individuals are male. Mitochondrial haplogroups were determined as H2a (Vittrup Man) and K1a3a (Svinninge Vejle). Both individuals carried Y chromosome haplogroup I2a.

Methods

We generated a dataset consisting of previously published European Hunter-Gatherers (HGs), Farmers and individuals associated with the Pitted Ware Culture on Gotland [56–58]. The included genomes from the Hunter-Gatherer and Farmer individuals were, in previous studies, shotgun-sequenced to a genomic coverage of > 0.1X and subsequently imputed. We obtained pseudo-haploid genotypes from the PWC and Danish Funnel Beaker Culture (FBC) associated individuals by first retaining sequencing reads with mapping quality of at least 30 and subsequently performing a random allele sampling. To avoid bias induced by combining imputed genotype data with pseudo-haploid genomes in the downstream analysis, we additionally generated pseudo-haploid genomes for every individual represented by imputed genotypes in our dataset. Finally, 108 Yoruban (YRI) individuals from the 1000 Genome project [59] were included in the dataset. We kept diallelic SNPs passing the 1000 Genome strict mask and imputation INFO score of above 0.5 (Bcftools v. 1.10.2) [60]. The final dataset included 403 genomes and 6.6 million variant sites.

The ancestry of the Vittrup and Svinninge Vejle individuals were investigated using principal component analysis (PCA) [61,62], model-based clustering [63] and D-statistics [64,65]. The PCA was carried out on a set of variants with a minor allele frequency (MAF) above 0.05 filtered using *plink v1.90b4.4 [66]*. We ran *smartpca* with the options *'lsqproject: YES'* and *'autoshrink: YES'* and computed PCs based on individuals with imputed genotype data onto which we projected the pseudo-haploid genomes. The ADMIXTURE analysis was carried out using only the pseudo-haploid genomes, restricting the analysis to SNPs with MAF of at least 0.05 and filtered sites based on linkage disequilibrium (LD) by pruning the data using *plink* (options: --indep-pairwise 1000 1000 0.1 --maf 0.05). The final dataset included 140,256 variant sites on which we ran a set of 20 repetitions for each K (2,3). We estimated the amount of excess allele sharing between Gotland PWC individuals and Early European Farmers relative to Swedish Hunter-Gatherers using D-statistics in the form of D(pop1, pop2; pop3, pop4). The test configuration represents the null-hypothesis stating that pop1 forms a clade with pop2 with respect to pop3. Standard errors and associated Z-scores were obtained with weighted block jackknife using a block size of 50 cM. We reject the null-hypothesis in cases where the Z-score > |3.3|. Resulting Z-scores below 0 suggest excess allele sharing between pop2 and pop3, while Z-scores above 0 suggest excess allele sharing between pop1 and pop3.

Results

PCA

We carried out a PCA to investigate the genetic ancestries of the Middle Neolithic Scandinavian HGs constituted by Vittrup, Svinninge Vejle, and the Gotlandic PWC individuals (in this SI chapter 'Scandinavian' means 'Swedish and Norwegian'). This way we explored their genetic relationship to the Mesolithic Western European and Scandinavian HGs, Eastern and Southern European HG groups as well as Early ('Anatolian') and Late Scandinavian/European Farmers (conventional genetic terminology). From the resulting PCA (Fig 7 and SI.6.1) we find PC1 separating HGs from Farmers, while PC2 separates Eastern European HGs from Western European HGs. The Farmers form a cline placing Early (Anatolian) Farmers furthest from the HGs followed by European Farmers and Scandinavian Farmers. Scandinavian HGs fall between the clusters of Western and Eastern European HGs suggesting that their ancestry is a mixture of these two categories [56,67,68].

Vittrup Man and the Svinninge Vejle individual cluster closely with the Mesolithic Scandinavian HGs and separate from the Middle Neolithic Scandinavian HGs associated with the PWC. The position of the PWC individuals along PC2 suggests that they share their East/West mixed HG ancestry with Vittrup and Svinninge Vejle, while they are slightly shifted from the Mesolithic Scandinavian HGs towards the Scandinavian Farmers along PC1 (Fig 7, for convenience also shown here as Fig SI.6.1). This indicates that the PWC individuals carry small amounts of Farmer ancestry due to admixture with Funnel Beaker

associated Farmers. A general scarcity of Stone Age human skeletal material from the Scandinavian Peninsula mainland is probably a primary reason for the current lack of Scandinavian Middle Neolithic individuals with a genomic profile similar to the humans from Vittrup and Svinninge Vejle.

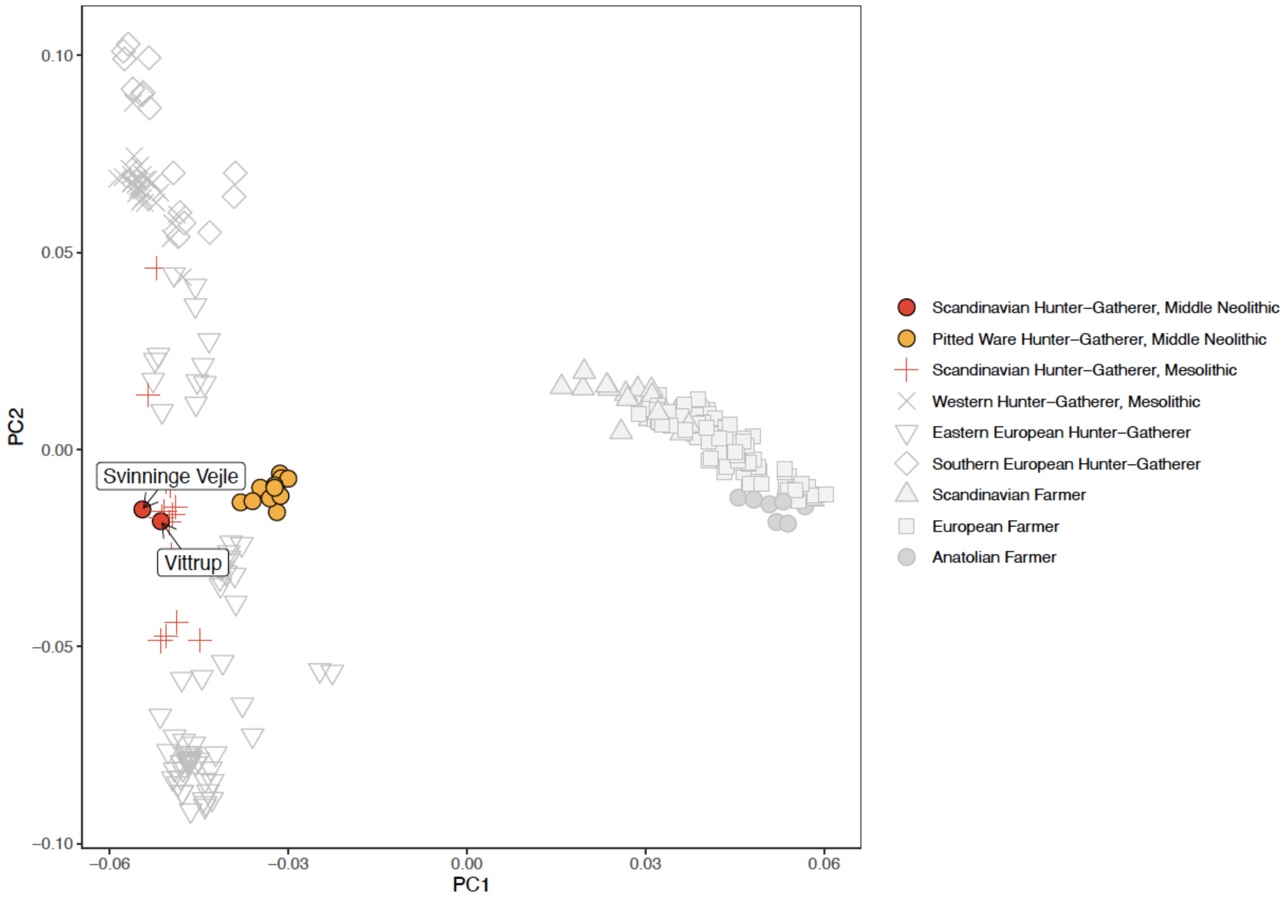

Fig SI.6.1. PCA, carried out by calculating PCs using only imputed genomes, onto which we projected the pseudo haploid genomes. Red dots represent the Middle Neolithic Scandinavian HG individuals from Vittrup and Svinninge Vejle. All PWC associated humans, known to us, are represented with orange dots. In the current genetic ancestry record of Mesolithic and Neolithic Europe, the humans most closely associated with Vittrup and Svinninge Vejle are Mesolithic age individuals from the Scandinavian Peninsula, here shown with +. The Pitted Ware Culture associated individuals from Gotland differ from the Vittrup and Svinninge Vejle males due to their genetic admixture with Funnel Beaker associated Farmers.

ADMIXTURE

As a second approach we use ADMIXTURE analysis to investigate and illustrate the ancestry proportions of the Vittrup and the Svinninge Vejle males. This is done across K=[2,3] in the context of the HGs and Farmers represented in the dataset. Vittrup and Svinninge Vejle are represented as Middle Neolithic Danish HGs while PWC is represented as Middle Neolithic Scandinavian HGs. From the results of K=2, we find the

first ancestry component (blue) maximised across HGs, while the second ancestry component (grey) is maximised among Farmer individuals. Thus, the majority of the ancestry of these two individuals is explained by the HG component. The PWC individuals draw most of their ancestry from the HGs and the rest from the Farmer component in the dataset (Fig SI.6.2)[56]. The small amount of grey in the K2 and K3 profiles for the Vittrup and Svinninge Vejle individuals could be an indication of an early admixture event between the ancestors of Vittrup and Svinninge Vejle and a local Farmer population. However, we cannot draw any conclusions on this only based on the ADMIXTURE analyses.

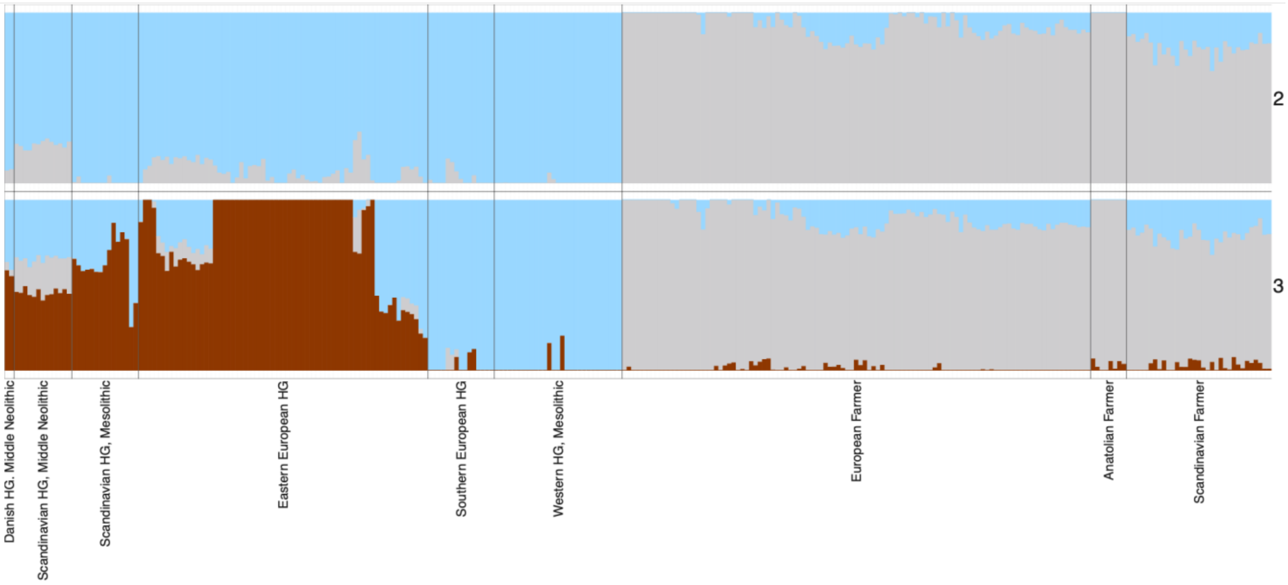

Fig SI.6.2. ADMIXTURE analysis using K=[2,3] in order to estimate ancestry proportions. Top = K2, bottom=K3. For the sake of simplicity, we label the Vittrup and Svinninge Vejle individuals 'Danish HGs, Middle Neolithic', due to the geographic location of their skeletal remains, which was possibly far distant from their respective places of birth. Individuals of Gotlandic PWC association are labelled 'Scandinavian HGs, Middle Neolithic'. The two ancestry components from K=2 are maximised in HGs and Farmers respectively, while K=3 components are maximised in Eastern European HGs, Western European HGs and European Farmers, respectively. We find the ancestry of Vittrup and Svinninge Vejle explained by the Eastern/Western European HG components, and the PWC ancestry as a similar mixture of HG components with an additional Farmer component fraction.

<u>*D-statistics*</u>

We carried out the D-statistics in form D(Sweden Mesolithic HG, X; Early European Farmers, YRI) to test for a significant number of shared alleles between the tested individuals and Early European Farmers not present in Swedish Mesolithic HGs. We carried out the test for Vittrup Man and Svinninge Vejle along with the Gotland PWC individuals. From the resulting Z-scores we find that neither Vittrup Man nor Svinninge Vejle

carry a significant level of Farmer ancestry. Their Z-scores are 2.1 and -1.3, respectively. We do however obtain formal evidence of excess allele sharing between the Gotland PWC individuals and Early European Farmers supporting the above findings (Fig SI6.3).

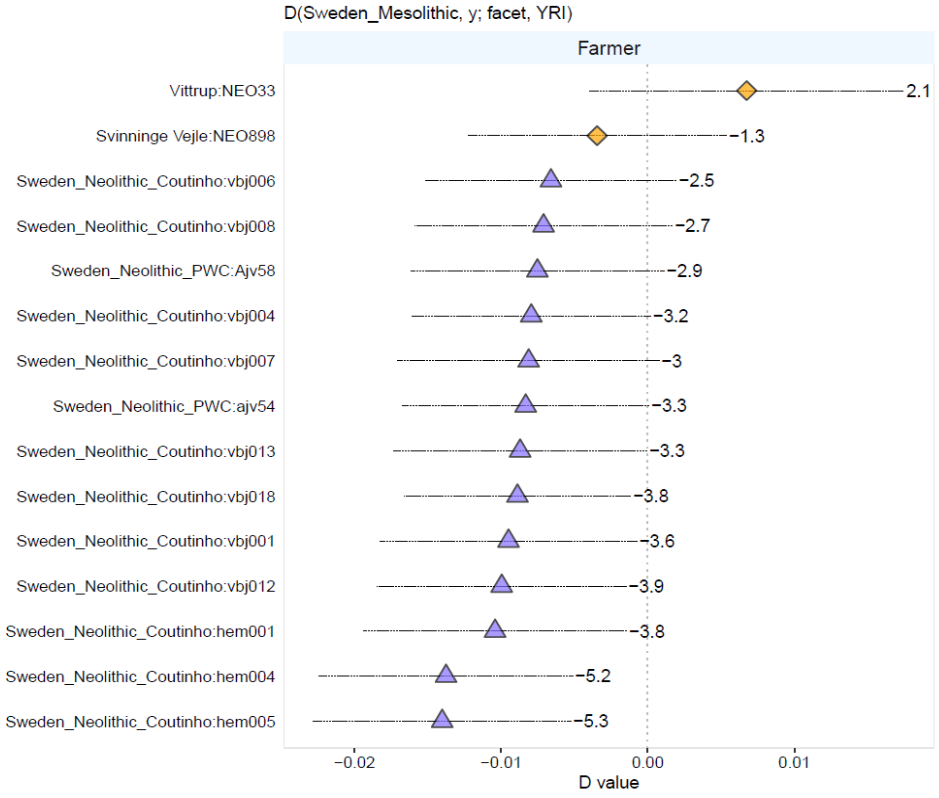

Fig SI.6.3. D-statistics in the form D (Swedish HGs, X; Farmers, YRI), where X represents Vittrup, Svinninge Vejle, or any of the PWC individuals. Each point indicates the D-value on the X-axis for an individual on the Y-axis. The associated Z-score is displayed next to the point, while the error bar represents 3×standard error. The resulting Z-score for both Vittrup and Svinninge Vejle is < |3.3|, meaning that we are not able to prove a significant amount of shared ancestry with the Farmers. For the majority of the PWC individuals, we are, however, finding formal evidence of Farmer admixture.

Conclusion

Based on the PCA, ADMIXTURE, and D-statistics analysis we find that Vittrup Man is closely related to the individual from Svinninge Vejle. In our comparison to HGs and Farmers, we find both of these Danish Middle Neolithic individuals to be closely related to the Scandinavian Mesolithic age HGs. The Gotlandic Neolithic age PWC individuals share their HG ancestry with the Swedish Mesolithic age HGs as well, although we find them to carry additional Farmer ancestry, due to a Funnel Beaker association, which we do not find significant evidence of in Vittrup Man or the Svinninge Vejle individual.

SI.7: Polygenic scores for the Svinninge Vejle individual

Alba Refoyo-Martínez, Andres Ingason and Fernando Racimo

Methods

The depth of genomic coverage of Vittrup Man is too low (<0.1X) for us to compute reliable polygenic score inferences. Instead, we studied the chronologically and genetically closely associated individual from Svinninge Vejle, as computed in Allentoft et al. [55] and Irving-Pease et al. [69]. The Svinninge Vejle individual, also a male, yielded ancient DNA with high preservation quality, and consequently higher genome coverage (~1X) so it was possible to obtain polygenic scores for various traits, including hair, eye and skin colour, height and basal metabolic rate (BMR). The latter is an estimate of the amount of energy that the human body needs to perform its most basic functions.

The polygenic score method is based on computing a weighted sum of effect size estimates for a given trait at approximately independent SNPs along the genome (reviewed in [70,71]). The weights in this score correspond to the genotype of the individual at those SNPs. The effect size estimates were obtained from a genome-wide association study (GWAS) performed on present-day inhabitants of the United Kingdom [72], Neale Lab GWAS: http://www.nealelab.is/uk-biobank], which was the closest high-powered panel to this individual that we could find among present-day GWASs. The genetic prediction of eye colour was done based on the HIrisPlex system [PMID:19278628]. Genotype likelihoods of all six main-effect SNP alleles were available in the ancient sample 1000G imputation, and imputed effect allele dosages of these were used to derive probabilities for brown, blue and grey/intermediate eye colour following the HIrisPlex formulas [PMID:19278628]. Computation of all these scores was carried out as part of the Allentoft et al. [55] and Irving-Pease et al. [69] studies.

The considerable distance in time and ancestry between the GWAS panel members and the Svinninge Vejle individual implies that polygenic trait inferences can only be made with large reservations, keeping in mind that differences in genetic architecture and linkage structure, as well as subtle patterns of stratification, may bias inferences of the genetic component of these traits in the ancient individual [73–76].

Results

We analysed the Svinninge Vejle genome to recover the genetic component of five traits that we deemed relevant because of their informative value in predicting the visual appearance and a gross metabolic characterisation of the ancient individual, and because their corresponding scores were highly over-dispersed across different ancient Eurasian ancient populations [55,69]. We obtained polygenic scores for the five traits in the Svinninge Vejle individual and compared the values of these scores with the values of the same scores

computed in other ancient Eurasian individuals for which we have genome-wide data (Figures SI.7.1 and SI.7.2). In Figure SI.7.2, we show scores for all available ancient West Eurasian individuals, while in main text Figure 9, we show scores for ancient Danish individuals [55,77–80].

At the time of the Vittrup and Svinninge Vejle individuals, two highly differentiated populations were present in Scandinavia: European Neolithic Farmers of Anatolian origin (associated with the Funnel Beaker Culture (FBC)) and Neolithic-age Scandinavian Hunter-Gatherers (associated with the Pitted Ware Culture). This separation can be observed using statistical methods that serve to summarise genome-wide patterns of population structure, such as principal component analysis (PCA) – a linear dimensionality reduction method often used for exploratory data analysis [81]. Along the first two PCA axes in Fig SI.7.1 the Svinninge Vejle male falls along the eastern-western hunter-gatherer ancestry gradient, along with other Scandinavian hunter-gatherer individuals from the region. He displays more typical hunter-gatherer phenotype scores than most other coeval individuals of the region (Fig 9, and Fig SI.7.2). This applies to:

- a higher probability of having had blue eyes (51%)
- relatively high score for dark hair
- relatively high score for dark skin pigmentation
- relatively high score for basal metabolic rate.

Unlike most of the other hunter-gatherer individuals, the height polygenic score for the Svinninge Vejle male falls well within the distribution of Neolithic individuals of Anatolian Farmer ancestry from the region, though not as high as the majority of Bronze Age and post-Bronze Age individuals. Overall, this suggests that he may have looked rather different from more recent and present-day Scandinavians, particularly in being relatively shorter and having darker skin and hair, if taking the polygenic score prediction at face value.

Compared with the contemporaneous FBC-associated individuals from Denmark, the Svinninge Vejle male may not have appeared remarkably different. As can be seen from Figure 9A, upper panel, the most prominent difference was probably a relatively darker skin colour. In terms of body height and darkness of hair, he appears to have been within the upper ranges of his local Anatolian contemporaries.

It is important to consider, however, that the polygenic scores of these traits only provide a partial picture, as the individual's environment, gene-by-environment interactions, differences in linkage and genetic architecture relative to the UK Biobank panel, and even other genetic loci that we are not including in the score may have played important roles in the true (realised) phenotype of this individual [71].

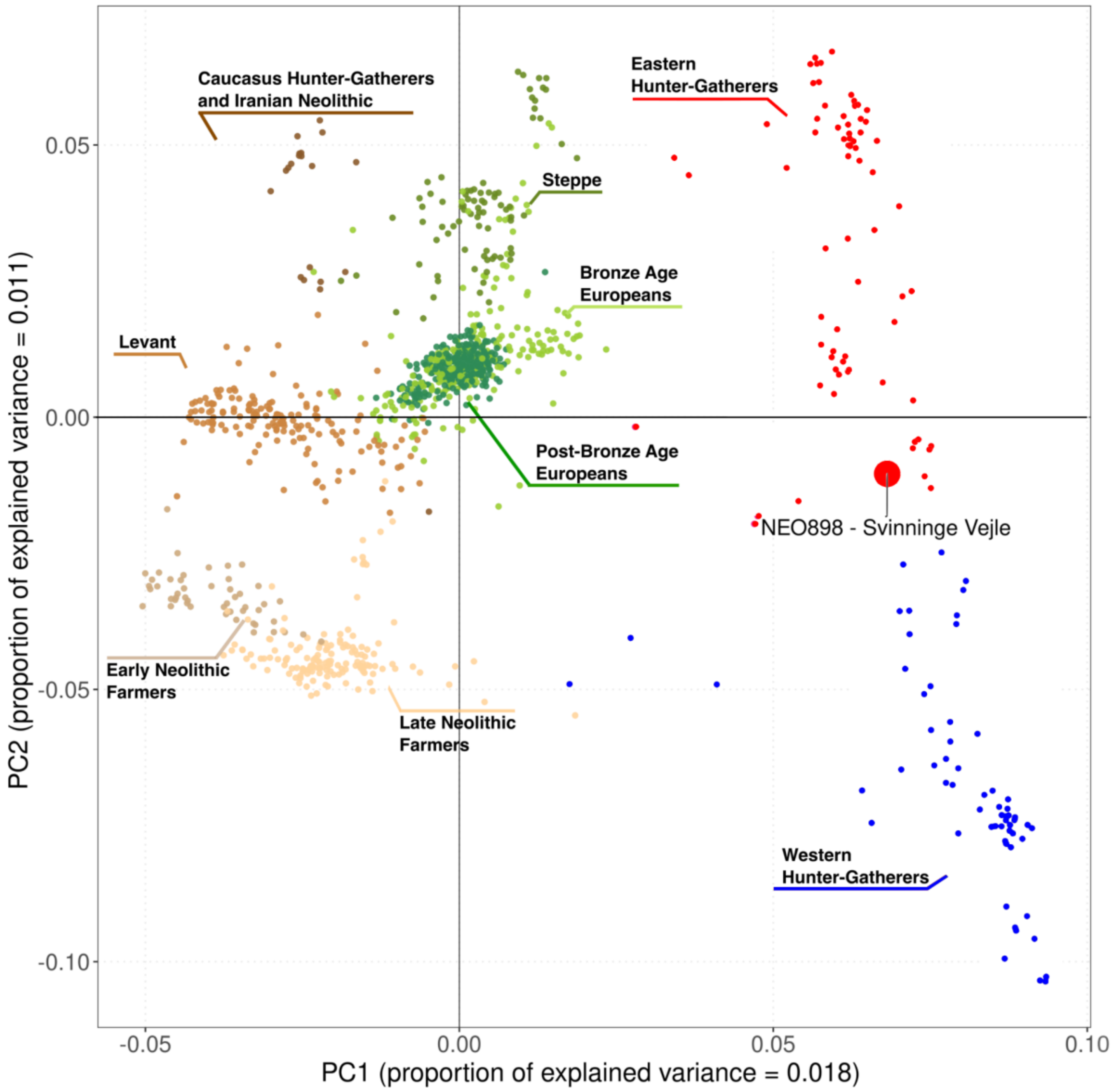

Fig SI.7.1. Principal component analysis (PCA) on 1164 ancient West Eurasian samples, coloured based on a clustering analysis from using tracts of identity-by-descent (IBD) [55]. We note that the IBD-based method captures finer structure than the first two PCA axes, which is why some groups are overlapping. The first principal component separates Asian, Steppe and European individuals. The second component separates West Eurasian Farmers and Hunter-Gatherers. Levant = Neolithic Farmers of the Levant. Early Neolithic Farmers = Early Neolithic Farmers of South and Central Europe. Late Neolithic Farmers = Later stage Neolithic Farmers, including Funnel Beaker Culture associates. Steppe = Late-stage Neolithic with Steppe ancestry.

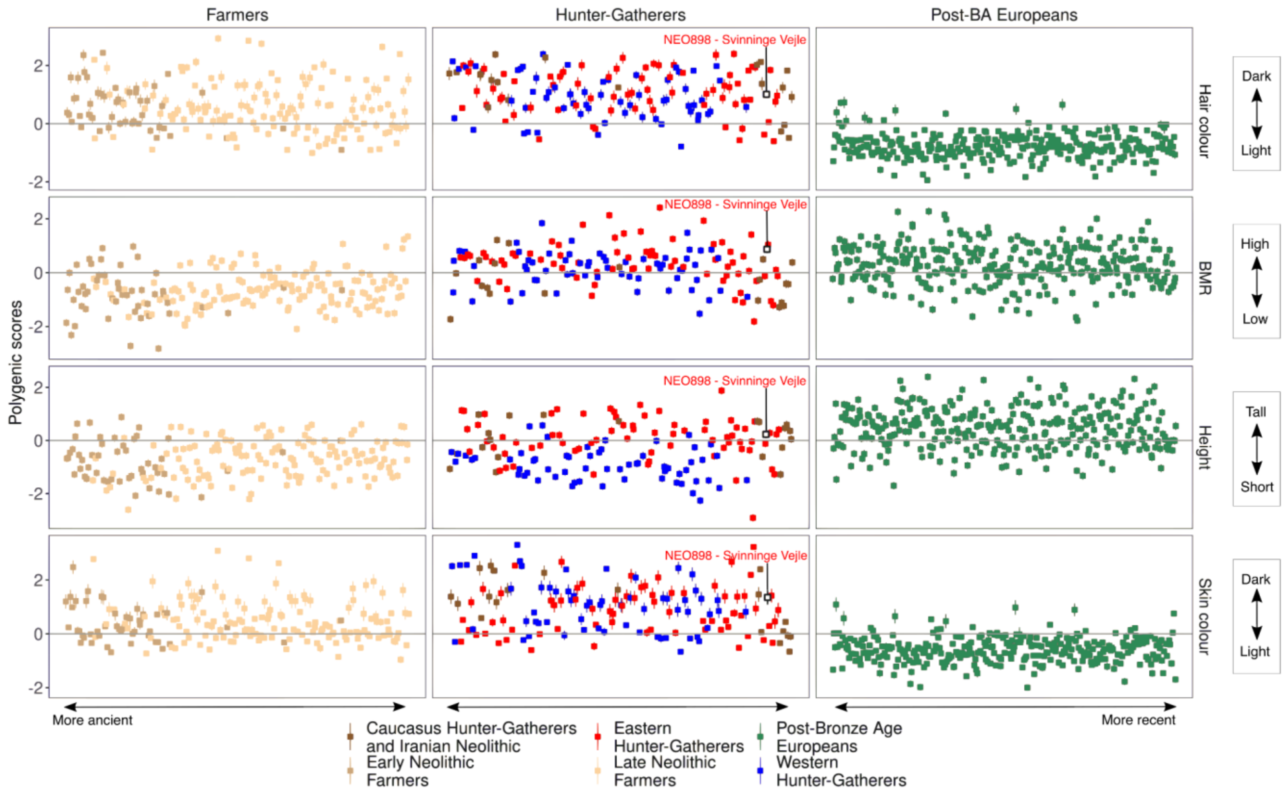

Fig SI.7.2. Polygenic scores for West Eurasian Neolithic Farmers, Hunter-Gatherers, and Post-Bronze Age groups, as computed in Allentoft et al. [55] and Irving-Pease et al. [69]. The groups are based on the genomes' placement on the PCA (Fig SI.7.1) and individuals are ordered by their chronological age. Polygenic scores for black hair, BMR (basal metabolic rate), height and skin colour by their population group. The Svinninge Vejle individual is depicted as an empty black square. The error bars denote 95% Bayesian credible intervals [74]. The samples are ordered along the x-axis in the same order as in Figure 9. The horizontal zero lines represent the global mean score across all samples.

SI.8: Enamel strontium, carbon and oxygen isotopes from Vittrup Man

Karl-Göran Sjögren, Anders Fischer and T. Douglas Price

Enamel from two of Vittrup Man's teeth were analysed for strontium, carbon and oxygen isotopes (Tables SI.8.1 and SI.8.2). This applies to his upper left first premolar and lower left third molar. Strontium isotope ratios ($^{87}Sr/^{86}Sr$) were obtained for the purpose of getting data on his geographical life-history, whereas the measurements of $\delta^{13}C$ and $\delta^{18}O$ values were produced for the purpose of dietary and environmental information relating to his childhood.

Table SI.8.1. Strontium isotope ratios in enamel from two of Vittrup Man's teeth.

**Species**	**Anatomical part**	**$^{87}Sr/^{86}Sr$**	**Lab. No.**	**Museum inventory No., etc.**
**Vittrup Man**				
Human, *Homo sapiens*	Upper left first premolar, (UP1; +4)	0.7134	F10741	VHM 54/1968C, 11600; AS 38/2018
Human, *Homo sapiens*	Lower left third molar (LM3; -8)	0.7159	F11090	VHM 54/1968C, 11600; AS 38/2018
**Vittrup aurochs and cattle, reference values**				
Aurochs, *Bos primigenius*	*Metacarpus*	0.7107	F10836	VHM 11599A
Aurochs, *Bos primigenius*	*Phalanx I*	0.7105	F10837	VHM 11599B
Cattle, *Bos taurus*(?)	*Metacarpus*	0.7098	F10838	VHM 11602A
**Krabbesholm 2, reference value**				
Red deer, *Cervus elaphus*	*Metacarpus*	0.7101	F11046	FHM4383; ZMK 50/2000, A1999; Poz-12127

The list includes reference values of roughly contemporaneous bones of terrestrial mammals from northern Jutland, including three specimens from the Vittrup mire. The risk of contamination from the depositional environment, applying to the bone samples, is considered unproblematic, since it will also be local. The sample from Krabbesholm, about 100 km to the south of Vittrup, is previously dated 5147±36 BP [35].
The measurements were conducted at University of North Carolina-Chapel Hill.

Our strontium analyses indicate a birthplace and early childhood in landscapes far from NW Denmark where Vittrup Man ended his life. In a European perspective, Sr isotope ratios similar to those found in his dentition are widely occurring. Since the DNA results suggest a geographic origin on the Scandinavian Peninsula or

neighbouring islands (SI.6), we focus on that region. Here Sr ratios matching those of Vittrup Man are found in several places, including SW Sweden and a coastal part of Norway close to the Arctic Circle [82–85]. Due to coarse sampling, it cannot be excluded that areas with equivalently high ratios can also be found further south along the Norwegian coast. The island of Gotland has lower baseline values and is, therefore, a less likely place of origin for this individual [86]. The higher value from his third molar as compared with the premolar suggests a change of residence before the age of 9–12 years. Since comparable variation in baseline values within restricted geographical areas are found in several places on the Scandinavian Peninsula, this change of residence need not have exceeded a hundred kilometres.

As opposed to isotopic signatures from bone collagen, enamel apatite $\delta^{13}C$ values reflect the composition of the whole diet, not only the proteins [87,88]. Enamel provides data only from early periods of life, varying depending on which tooth is analysed. The crown of the premolar tooth is formed during years 2–6, while the third molar crown is normally formed from the age of 9–10 to the age of 13–14 years (SI.2).

Ambrose and Norr [89], based on feeding experiments with rats, found that when the protein and bulk diet have the same $\delta^{13}C$ values, apatite is enriched by 9.4‰ relative to the total diet. Harrison and Katzenberg [87], on the other hand, found a $\delta^{13}C$ apatite-diet offset of +12.0‰ more accurate for human samples. Tykot et al. [88] also argue for an enrichment of about 12‰ based on the evaluation of other human apatite and enamel values. For ruminants, even higher values have been reported, up to c. 14‰ for cattle [90]. From Table SI.8.2 it appears that an additional offset factor should possibly be suspected between $\delta^{13}C$ and $\delta^{18}O$ values from unpretreated versus pretreated samples. However, offsets of the scale seen in the table will not affect our inferences below.

Normal enamel $\delta^{13}C$ values in Neolithic humans from northern Europe are around -14‰ [91], corresponding to a dietary mean of c. -26‰, using the 12‰ offset. This would be the expected value for humans subsisting mainly on terrestrial animals (meat and/or milk products) and cultivated plants. The value from the premolar of Vittrup Man is considerably higher, at -10.5‰, suggesting a mean dietary value of c. -22.5‰. The $\delta^{13}C$ value from his lower left third molar, on the other hand, is c. 4‰ lower and is similar to other Neolithic individuals from northern Europe (Table SI.8.1). Consequently, a change in diet must have occurred between the formations of these two teeth, i.e. between the ages of c. 10 and c. 13–14 years.

The enamel $\delta^{13}C$ values obtained from Vittrup Man's teeth probably result from a mix of several different components with differing isotopic signals. However, the high enamel $\delta^{13}C$ values in the premolar suggest a more important contribution of food from marine sources during childhood than during his teenage life.

The $\delta^{18}O$ values measured in the premolar and third molar depend ultimately on climatic conditions at the time of tooth formation. $\delta^{18}O$ is often used in environmental studies as a proxy for climate but has in archaeology also been used as a proxy for geographical movement. Apart from mountainous regions, oxygen varies only over quite large geographical scales, and is also subject to substantial seasonal and short-term fluctuations. Further, baselines of $\delta^{18}O$ variation in prehistoric times are poorly known. These problems hamper our possibilities to use $\delta^{18}O$ for mobility studies, but they can still be discussed as indications of possible movement, for instance by comparing different samples from one and the same individual or by comparing values from one individual with other individuals from the same region.

In the case of Vittrup, the value measured on the premolar is lower than most measured values from Neolithic Danish individuals, while the value from the third molar is close to the mean of these values (-4.42±0.99‰; Rise project, unpublished data). This could indicate that he spent his early years in a colder region than Neolithic Denmark. It should, however, be pointed out that the premolar value is not different enough from the overall mean to securely exclude short term climatic variation as an explanation.

Table SI.8.2. Carbon and oxygen isotope ratios from the enamel of Vittrup Man's upper left first premolar (+4) and lower left third molar (-8).

Tooth	Pretreatment	$\delta^{13}C$ VPDB	Std dev	$\delta^{18}O$ VPDB	Std dev	Voltage	Sample ID
PM (+4)	Unknown	-10,46	-	-5,91	-	-	F10741
LM3 (-8)	None	-13,72	0,017	-4,29	0,095	1,47	F11090/K1341
LM3 (-8)	None	-13,41	0,005	-3,49	0,022	2,00	F11090/K1341
LM3 (-8)	Acetic acid	-14,49	0,038	-4,63	0,035	1,98	F11090 PT/K1341
LM3 (-8)	Acetic acid	-14,36	0,034	-4,49	0,035	1,27	F11090 PT/K1341
LM3 (-8)	Average (PT)	-14,43		-4,56			

The powdered enamel of M3 was divided into two parts, one was untreated and one bathed in acetic acid (0.1 ml for 1 hour). Each part was measured in duplicate. Analytical precision (1 sigma) ± 0.08 for $\delta^{13}C$ VPDB, ± 0.10 for $\delta^{18}O$ VPDB. Average values from the pretreated sample (PT) are used in the discussion. The measurements were conducted at University of Arizona, Tucson.

We are aware that further isotopic analyses of teeth formed relatively early in Vittrup Man's life will potentially add significantly to the understanding of dietary and geographic aspects of early stages of the life-history of this very special individual. Anyhow, we have for research ethical reasons, decided not to conduct additional destructive sampling (cf. SI.4).

SI.9: Bone collagen $\delta^{13}C$ and $\delta^{15}N$ dietary signatures for Vittrup Man

Karl-Göran Sjögren, Darren R. Gröcke and Anders Fischer

As part of the present project several sets of data have been produced for the sake of reconstructing Vittrup Man's dietary life-history (SI Chapters 8, 9, 10, 11). In this chapter we present and discuss two sets of data, produced in Belfast and Durham, respectively. They concern bone collagen measurements, based on skeletal elements of Vittrup Man and on bones of food source mammals geographically and chronologically closely associated with this human.

In addition to dating, collagen stable isotopes in four samples from Vittrup Man's skeleton were analysed at the Chrono Centre at Queen's University Belfast (Table 1, main text). Two of these derived from different portions of the hard palate and other two were taken from cortical parts of a shin bone (*tibia)* and an ankle bone (*talus*) (main text Fig 3 and Table 1). Their $\delta^{13}C$ and $\delta^{15}N$ isotope ratios were determined using a ThermoScientific Delta V Advantage IRMS coupled to a Thermo FlashEA 112 Series Elemental Analyser.

Collagen extraction methods utilised in Belfast have been described by Reimer et al. (2015). Collagen was extracted using standard protocols based on a modified Longin method [92–95]. Ultra-filtering was applied in all four cases. Measurement precision for $\delta^{13}C$ and $\delta^{15}N$ were 0.22‰ and 0.15‰, respectively, which is within the generally accepted analytical error of ±0.2‰ (1sd). Likewise, all are within the acceptable atomic C:N range, and therefore show a low likelihood of diagenesis and/or contamination [96,97].

Four bone samples from the Vittrup site were selected for dietary isotopic analysis at Durham University. One of these came from Vittrup Man's hard palate (*palatine*) - once again from a different portion of the hard palate. The other three were bones of the genus *Bos* sp. (Table SI.9.1) which were found close to the human remains (SI. 5). AMS dating of all four specimens demonstrate that they are of roughly coeval age and are deposited within the period c. 3600–3300 cal BC (Table 1, and SI.1).

The bone collagen dietary analysis conducted in Durham partly duplicated what was received from the AMS dating lab at Belfast University (Table 1, main text). The primary reason for this duplication of analyses was to generate an internally comparable and consistently obtained set of isotope values, to be made use of in combination with an incremental dietary analysis of one of Vittrup Man's teeth (SI.10).

Sample preparation took place in the Stable Isotope Laboratory in the Department of Archaeology at Durham University. Specimens were first cleaned of any obvious surface contamination with a dental burr, after

which they were demineralized utilising a standard Longin method [92–94]. Extracted collagen was subsequently analysed in the Stable Isotope Biogeochemistry Laboratory (SIBL) located in the Durham University Department of Earth Sciences. Results are presented in Table SI.9.1.

Table SI.9.1. Carbon and nitrogen dietary isotopic values for Vittrup Man and reference values measured on bone collagen from geographically and chronologically closely associated food source animals.

**Material sampled**	**Taxon**	**$\delta^{13}C_{VPDB}$ (‰)**	**$\delta^{15}N_{AIR}$ (‰)**	**C:N**	**Collagen yield (%)**	**%N**	**%C**	**Inventory no**	**Lab code**
**Vittrup Man**									
Maxilla (palatinum)	Human *(Homo sapiens)*	-20.6	10.0	3.4	21.1	15.8	45.5	VHM 11600C	4020
**Reference values for terrestrial herbivores**									
Metacarpus	Aurochs *(Bos primigenius)*	-23.0	4.8	3.3	22.8	15.9	44.6	VHM 11599A	4760
Phalanx I	Aurochs *(Bos primigenius)*	-23.3	5.0	3.4	22.8	15.8	43.6	VHM 11599B	4761
Metacarpus	Domestic cattle (?) (*Bos taurus* (?))	-21.8	5.4	3.5	18.3	14.8	44.1	VHM 11602A	4762

All values are averages of duplicate measurements, conducted in Durham.

The two sets of $\delta^{13}C$ and $\delta^{15}N$ isotope ratios - from Belfast and Durham, respectively - are mutually consistent with the measuring uncertainties pertaining to them.

Bone collagen $\delta^{13}C$ and $\delta^{15}N$ ratios primarily reflect protein sources [89,98]. A closer understanding of an individual's dietary habits requires background knowledge of the isotopic characteristics of the potential food sources at the time and the place where they lived. From Stone Age Denmark and southern Sweden such reference values have already been established for multiple terrestrial, marine, and freshwater taxa,

as well as for domestic cereals [35,99–107]. The reference material can now be supplemented with data for the three bones of *Bos*, found at the Vittrup site.

The isotopic signature for the protein component of Vittrup Man's diet suggests a dominant role for terrestrial food sources such as cultivated and wild plants, domestic and wild animals. Since the isotope values for cereals of the period in question are similar to those of contemporaneous domestic cattle, probably due to manuring [35,106,108,109], we refrain from estimating the relative proportions of plant versus animal foods. Likewise, the difficulties separating individual foodstuffs of the same ultimate source (e.g. cattle-derived meat vs. milk products) makes it difficult to determine the relative contribution of sources, which from other forms of evidence seem to have contributed to human diet during this part of the Scandinavian Stone Age [110,111].

The dietary isotope values of Vittrup Man's bone collagen fall within the known prehistoric Danish terrestrial range, at the higher end of the range's $\delta^{13}C$ and $\delta^{15}N$ values [35]. As bone collagen reflects a dietary average over a number of years, this implies that the individual's diet was either tightly controlled within this range, meaning relatively little dietary diversity (cf. Fig 12 and comments to this in the main text), or the observed value is the result of a more diverse diet consisting of nutrients which included primarily terrestrial foods, but also marine protein. The latter may be closer to reality, given that fishing during the Funnel Beaker epoch in Denmark is indicated by finds of large fish weirs [112] and more directly by the presence of marine lipid signatures in pottery [113].

SI.10: Dentine δ^{13}C and δ^{15}N dietary isotope profiles of Vittrup Man

Darren R. Gröcke and Anders Fischer

The lower left third molar (wisdom tooth) from Vittrup Man (Fig SI.10.1) provides us with information on variation in this individual's life-way during late childhood and teenage, i.e. between c. 12–14 and c. 19–20 years of age (SI.2). This is due to the nature of dentine formation, allowing for a rough temporal resolution, based on sequential sub-sampling of small horizontally sectioned dentine increments from cusp to root apex [114,115]. Since no 'growth-rings' are observed and due to the only partially horizontal nature of dentine growth, the isotopic changes seen in main text Figure 12 should probably be understood as the somewhat smoothed out signals of the actual changes in life-conditions experienced by this human. The variations through time of his dentine δ^{13}C and δ^{15}N values primarily reflect changes in diet, whereas the basic reason for the trend towards rising δ^{34}S ratios most likely represent change in geography. Changes in physiological stress may, however, also be represented in the isotopic values [92–94].

The tooth was first halved along a lingual-buccal axis and excess enamel removed. The half that was made available for the present analysis was then demineralised in 0.5M HCl. During the demineralisation process the top portion of the tooth (the enamel underlying what was the crown) detached from the rest of the tooth, making it unclear as to the original orientation of this segment (e.g. which way was up). This detached portion and the remainder of the tooth were then cut into 16 total increments, but with the first four (4328-1 to 4328-4) in insular unclear chronological order albeit earlier in life than the remaining 12 (4328-5 to 4328-12). From these increments dentine collagen was isolated using a modified Longin method [92–94] in the Department of Archaeology at Durham University as detailed in SI.9. Extracted collagen increments were analysed in the Stable Isotope Biogeochemistry Laboratory (SIBL) in the Department of Earth Sciences at Durham University following the protocols given in [116] (Appendix I). Data are given in Table SI.10.1, and a synthesis is presented in the main text.

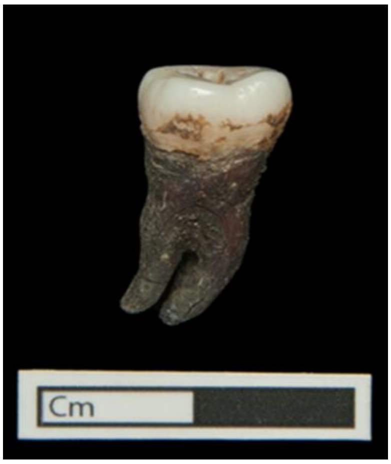

Fig SI.10.1. The dentine (dark part) of this wisdom tooth of Vittrup Man's was cut up horizontally in c. 1mm slices. Dietary isotopic analysis of these reveals fundamental changes in his life-way at the end of his teenage years. Photo: Durham University, Jeff Veitch.

Table SI.10.1. Incremental dentine collagen carbon and nitrogen isotope ratios from Vittrup Man's lower left wisdom tooth.

**Sequential sample no.**	**%N**	**$\delta^{15}N_{AIR}$ (‰)**	**%C**	**$\delta^{13}C_{VPDB}$ (‰)**	**C:N atomic**	**Lab. No.**
Increment 1	n.d.	n.d.	n.d.	n.d.	n.d	4328-1
Increment 2	~~11.9~~	~~10.7~~	~~42.2~~	~~-20.4~~	~~4.1~~	4328-2
Increment 3	13.2	11.2	40.6	-20.5	3.6	4328-3
Increment 4	14.2	11.4	41.5	-20.8	3.4	4328-4
Increment 5	14.8	11.5	42.2	-20.2	3.3	4328-5
Increment 6	14.9	11.0	41.7	-20.3	3.3	4328-6
Increment 7	14.6	11.2	43.2	-20.5	3.5	4328-7
Increment 8	14.6	11.3	42.8	-20.6	3.4	4328-8
Increment 9	14.8	11.4	42.0	-20.2	3.3	4328-9
Increment 10	15.0	11.6	43.1	-20.0	3.4	4328-10
Increment 11	14.8	11.4	42.2	-20.1	3.3	4328-11
Increment 12	14.9	11.3	42.9	-20.2	3.4	4328-12
Increment 13	14.8	11.6	42.7	-20.0	3.4	4328-13
Increment 14	14.7	11.9	42.2	-19.9	3.3	4328-14
Increment 15	14.6	11.2	42.4	-20.2	3.4	4328-15
Increment 16	14.3	10.3	43.5	-20.7	3.5	4328-16

Struck-through values have unacceptable atomic C:N ratios. The tooth in question has the lab id no. SIBL 4328.

SI.11: Sulphur isotope analysis of Vittrup Man

Darren R. Gröcke and Anders Fischer

Measurements of human bone collagen sulphur isotope ratios (δ^{34}S) have the potential to provide information of relevance to dietary reconstruction, etc. [117]. δ^{34}S values (Table SI.11.1) for three roughly contemporary *Bos* sp. specimens recovered near to the Vittrup individual (main text Table 1; Fig SI.1.6), may in this regard provide important base-line data. Therefore, δ^{34}S analyses were run at bone samples from Vittrup Man and the three bones of *Bos* sp. Analyses were performed at Durham University on the very same bone collagen extracted per the protocols detailed in SI.9. The samples were then analysed in the Stable Isotope Biogeochemistry Laboratory (SIBL) within the Department of Earth Sciences at Durham University using the methods presented in [116]. Results are detailed in Table SI.11.1.

One of the bovine samples (Lab #4762) produced unacceptable atomic ratios of C:S and N:S [118], however the Vittrup individual falls within the ±10% range of acceptable values proposed by Nehlich & Richards [118]. The two *Bos primigenius* specimens had collagen of acceptable quality and produced δ^{34}S values of 12.2‰ and 1.8‰. The slight marine signature in these values may reflect proximity to a marine habitat [117] and/or an underlying marine-deposited geology, in the landscape where they lived out their lives. Both conditions were in fact met in the surroundings of the Vittrup site (SI.1). The Vittrup individual has lower δ^{34}S values than the auroch samples. A paucity of geographically and chronologically relevant δ^{34}S baseline values from food species potentially consumed by Vittrup Man prevents us from presenting a solidly based interpretation of these results. Potentially, we may have another (cf. SI. 6, 8 and 10) indication that this human did not spend all of his life in the Vittrup area.

Table SI.11.1. δ^{34}S collagen data for Vittrup Man and three nearly contemporaneous Bos. *sp specimens.*

**Species**	**Bone**	**%S**	**δ^{34}S (‰)**	**Atomic C:S**	**Atomic N:S**	**Collagen yield (%)**	**%N**	**%C**	**Atomic C:N**	**Museum inventory no.**	**Durham Lab. No.**
Homo sapiens	Maxilla (palatinum)	0,4	8,3	320	97	21,1	16,5	46,9	3,3	VHM 054/1968 x11600C	4020
		0,4	8,8	308	91	21.1	15,2	44,0	3,4		4020
Bos primigenius	metacarpal	0,3	12,2	400	122	22.8	15,8	44,6	3,3	VHM 11599A	4760
Bos primigenius	phalanx 1	0,3	11,8	347	103	22.8	15,1	43,6	3,4	VHM 11599B	4761
Bos taurus (?)	Metacarpal	~~0,5~~	~~10,3~~	~~232~~	~~67~~	18.3	14,8	44,1	3.5	VHM 11602A	4762

The sulphur measurement, based on the human palatine bone, was run in duplicate. Struck-through values have atomic C:S and N:S values outside the acceptable ranges.

SI.12: The Svinninge Vejle individual

Anders Fischer, Marie Louise Jørkov, Ole Bennike, Anne Birgitte Gotfredsen and Karl-Göran Sjögren

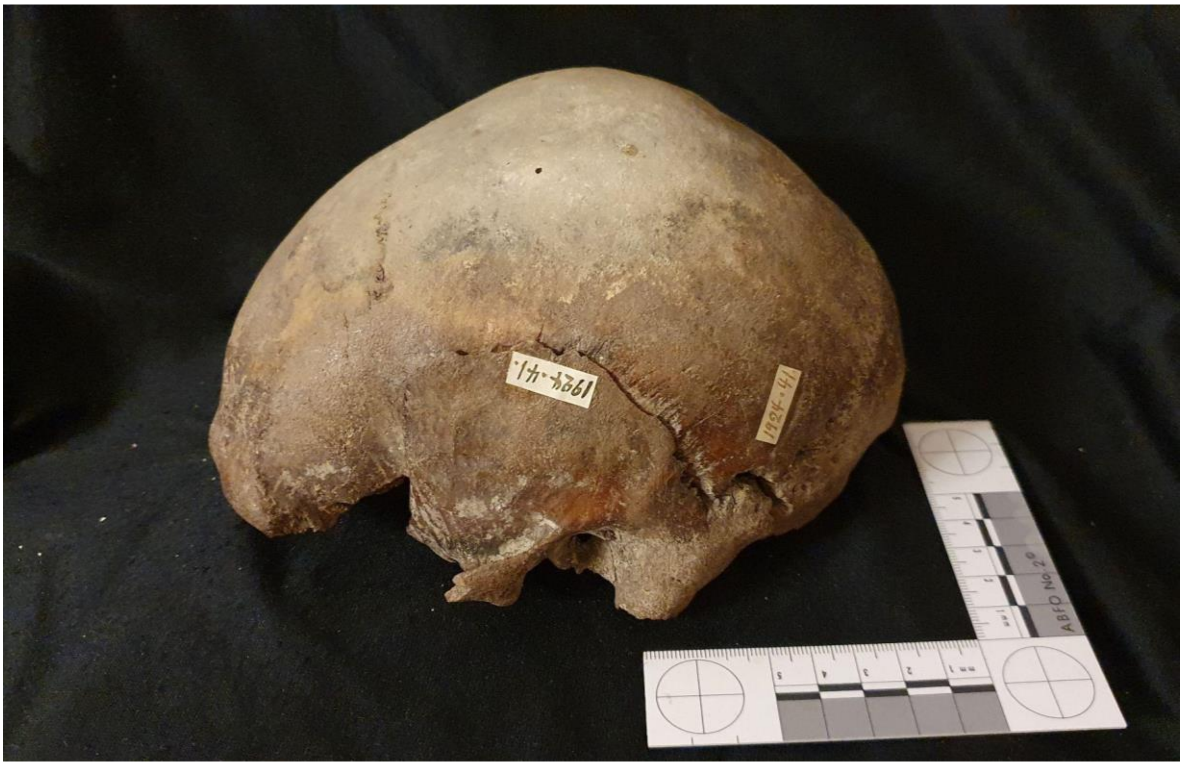

Fig SI.12.1. A calvarium of an adult male found in marine sediments of Svinninge Vejle, Denmark. Photo: ML Jørkov.

All that is available of the Svinninge Vejle individual is a fragment of a human calvarium (Fig SI.12.1). It was submitted in 1929 to the Geological Survey of Denmark (DGU, now GEUS) together with cranial remains of a relatively large whale (Cetacea sp., most likely a baleen whale) by medical practitioner Ejnar Theodor Tulinius from the nearby village of Snertinge. The rather scarce provenience data (see below) indicated a Stone Age date for the calvarium. This judgement has been confirmed via an AMS analysis, arranged as part of the genomic-archaeological project [55] of which the present paper is a spinoff. The analysis showed the bone collagen to be well-preserved and proved the individual in question to be of Neolithic date (Table SI.12.1).

The calvarium is of an adult male. Its vault bones are remarkably heavy and dense, and measure between 7.2 and 9.5 mm in thickness. Muscle attachments are similar to but not as pronounced as those seen in Vittrup Man. Patina and sediment depositions indicate that the skull has been fragmented since prehistoric times. Contrary to the Vittrup case there are, however, no signs of deadly violence as concerns the skeletal remains available.

The find spot for the whale bones was examined on 4 June 1929 by geologist Sigurd Hansen of DGU, and based on circumstantial evidence it may be assumed the human remain was found nearby or right next to it (Fig SI.12.2). Anyhow, its physical appearance makes likely the latter skull fragment derives from a marine sediment. According to Hansen's field notes the whale remains were found in a small pit where marine mollusc shells in the shell-rich sediment were extracted for commercial use.

The bones were found at 55.738°N, 11.436°E, at 0.7 m below mean sea level, in the middle of the reclaimed fjord Svinninge Vejle. During the following months further study of the stratigraphy took place, and the elevation of the find was determined (Fig SI.12.3). At the bottom clayey sand was found, interpreted as Late-glacial. The sand was overlain by clayey gyttja and detritus gyttja, followed by peat. Next followed a shell-rich layer with many shells of small cockles (*Cerastoderma* sp.), oysters (*Ostrea edulis*) and carpet-shell (*Tapes* sp.). This was overlain by marine gyttja with shells of cockles, another shell-rich layer dominated by cockles and without shells of oyster or carpet-shell and finally a soil layer. Bones were found in the lower shell-rich layer, at 10–50 cm above the boundary between peat and shell-rich sediment. Judged on the basis of local geological data (Bennike et al. 2020) the lower shell-rich layer from Svinninge Vejle is probably of Mid-Holocene age – agreeing with the AMS date for the calvarium (see below).

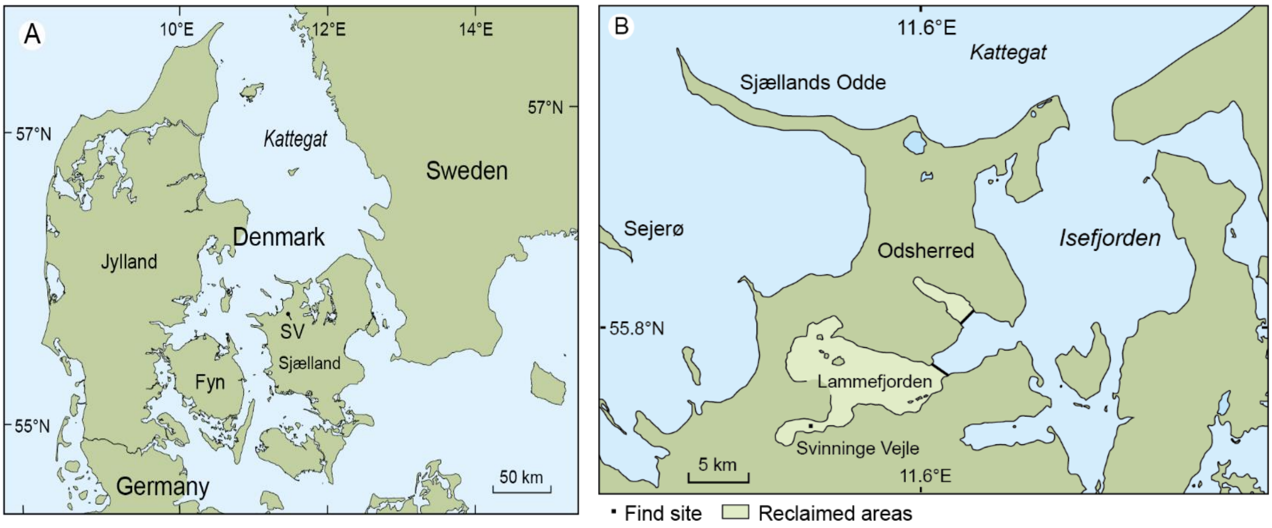

Fig SI.12.2. The location of Svinninge Vejle in NW Sjælland, Denmark (A), and the extent of former fjords, reclaimed from the 1870s onwards (B). Graphics by Ole Bennike/GEUS, based on map data from the Danish Geodata Agency.

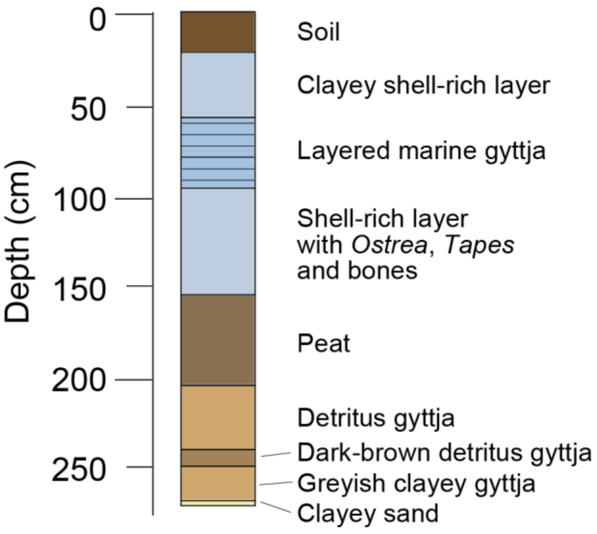

Fig SI.12.3. Stratigraphy at the find site for whale bones (and possibly also the human calvarium), based on notes by Sigurd Hansen, kept in the archives of the Geological Survey of Denmark and Greenland.

The find site for the calvarium has been below sea-level since the Mesolithic [119]. During the days of deposition of the bone, the local relative sea-level was about 3 metres higher than today [12].

Except for Vittrup Man, the Svinninge Vejle individual is the only one from Denmark that has a genetic profile different from all other analysed skeletons of the Middle Neolithic Funnel Beaker epoch. Genomically their closest associates are Mesolithic age individuals from mainland Norway and Sweden (SI.6). Regrettably, we cannot use the Sr method to determine if his region of birth was also outside present-day Denmark. Such an analysis would have demanded a sample from the innermost parts of the petrous bone, all of which has, however, during an early stage of the study been used for DNA analysis, AMS dating and measurement of $\delta^{13}C$ and $\delta^{15}N$ values. The Sr value of 0.709578, measured on left-over bone material from the outer part of the *pars petrosa*, is within the typical range for Danish FBC associated individuals. If Svinninge Vejle Man had subsisted on typical FBC diet, this value could indicate he as an adult may have lived in Denmark. Not knowing the $\delta^{13}C$ and $\delta^{15}N$ values for the Sr measured sample, we cannot however, exclude it simply reflects a predominantly marine diet (the marine Sr value is approximately 0.7092 [120,121]). Nor can we exclude that the porous bone sample has been affected by contamination from the burial environment.

The $\delta^{13}C$ and $\delta^{15}N$ values of the Svinninge Vejle male also differ from all other Danish skeletons of Middle Neolithic date in our genome study [55] (main text Fig 11). Human skeletons of roughly coeval date with related isotope signatures are known from the east-coast of Sweden and from the Baltic islands

Gotland and Öland [99,101]. They belong to the archaeological find complex called Pitted Ware Culture. His δ^{13}C value suggests a substantial contribution of protein from marine sources. If he lived in a Danish or West Swedish region, the proportion of marine versus terrestrial protein can be roughly estimated to c. 35%, but if he lived in a brackish environment such as the Baltic Sea region, the proportion could be even higher. The δ^{15}N value also supports a contribution from protein sources at a high trophic level, such as fish or marine mammals. Taken together these dietary isotopic observations may be taken as indications of the Svinninge Vejle individual being a first-generation immigrant - as is demonstrated in depth with Vittrup Man.

Table SI.12.1. ^{14}C date, dietary isotope values and preservation quality parameters for the Svinninge Vejle individual.

**Material dated**	**^{14}C years BP**	**Reservoir corrected cal age BC (2σ)**	**δ^{13}C (‰ VPDB)**	**δ^{15}N (‰ AIR)**	**C:N**	**Collagen %**	**Lab. No**	**Inventory No.**
Left *pars petrosa*	4539±72	3341-2921	-17.1	14.2	3.17	9.80	UBA-37912	AS 9/93; ZMK 41/1941

To estimate marine reservoir effect, the degree of marine protein intake was calculated as a linear interpolation between a marine and a terrestrial endpoint. Endpoint values of δ^{13}C -21.7 to -10.1‰ were originally measured on coeval local fish and terrestrial fauna. For the sake of stressing uncertainties, we here use the rounded end values of -21‰ and -10‰. In addition, we assume that the reservoir effect in the marine environments of the relevant region and period was 273±18 years [122]. No ΔR value is applied.

When corrected for marine reservoir effect, the radiocarbon dates for the Svinninge Vejle and Vittrup individuals overlap each other. The former, however, has a 95.4% probability range that ends significantly later (Fig SI.12.4). This may lead to different judgments as to these two individuals' relation to the Pitted Ware habitation remains in Denmark, which represent cultural influence and perhaps immigration from the Scandinavian Peninsula. Settlement materials referable to this archaeological group are most pronouncedly represented on the peninsula of Djursland, where it occurred between c. 3100–3000 cal BC [123,124]. Based on the undisturbed stratigraphy obtained from the Ginnerup site on Djursland, combining radiocarbon dates and culture-specific artefact types, the Pitted Ware Culture in Denmark most likely followed or slightly overlapped with the Vittrup and Svinninge Vejle individuals [125]. Especially for the latter, an overlap in time is probable (Fig SI.12.2).

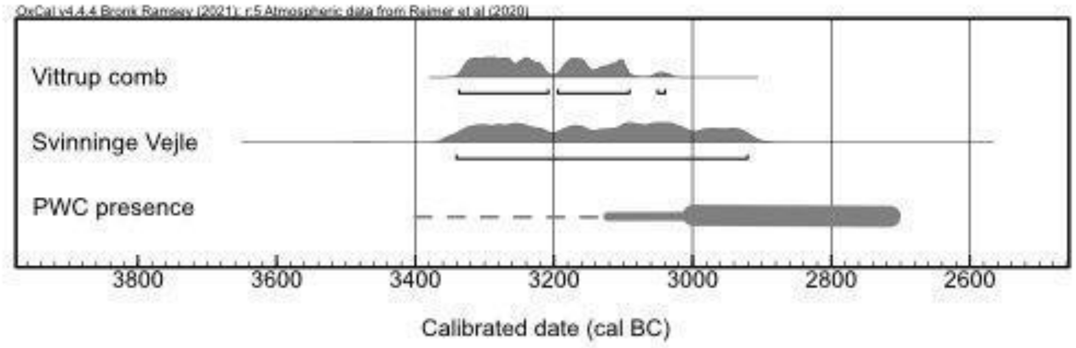

Fig SI.12.4. Probability graph for the combined AMS dates for the Vittrup and Svinninge Vejle individuals, respectively, after reservoir correction. Also shown is the estimated time range for the Pitted Ware Culture on Djursland.

The deposition of the Svinninge Vejle individual in a marine environment is not unusual as compared with contemporaneous skeletons from Denmark. Although most Funnel Beaker epoch human skeletal remains derive from burials and bogs, there are two other AMS dated and genetically profiled human remains found in the sea-floor: Roskilde Fjord and Stenderup Hage [55]. In these cases, as with most FBC period human bog skeletons, there are no signs of deadly violence, although other kinds of evidence indicate these individuals were brought involuntarily to the watery sites in question and were killed there e.g. [126].

The Svinninge Vejle skull fragment now belongs to the Laboratory of Biological Anthropology, Copenhagen University, where it is catalogued under no. AS 9/1993.

SI.13: Neolithic material culture demonstrates reciprocal connections between western Norway and north-west Denmark

Lasse Sørensen

Vittrup Man belongs within the time interval 3368-3104 cal BC (95.4% probability interval). According to other parts of this study, his childhood home was in a coastal region of the Scandinavian Peninsula, where the climate was apparently colder than Denmark was at the same time (SI 6 and 8). The question is whether the geographical area of his childhood home can be identified? By exploring Scandinavian archaeological records, it will be demonstrated in the following that a realistic option is western Norway.

The museum collections in Denmark and Norway contain various kinds of evidence, which indicate the existence of exchange networks, through which objects and materials were transported from the vicinity of Vittrup in Vendsyssel, the north-westernmost part of Denmark, to more northern parts of Scandinavia, and *vice versa*. One important find that is possibly associated with this network is a funnel beaker with vertical belly stripes from the second half of the Early Neolithic, which was fished up from the seabed at a depth of c. 120 m, 5–7 nautical miles (9–13 km) north of Skagen, the present-day northern tip of Jutland, NW Denmark (Fig SI.13.1) [17,127]. Around 20 km have been added to the spit of Skagen since the ceramic vessel sank to the bottom of the sea roughly 3500 cal BC [128]. The funnel beaker can therefore be interpreted as having ended up at this location during a voyage on the open sea [129].

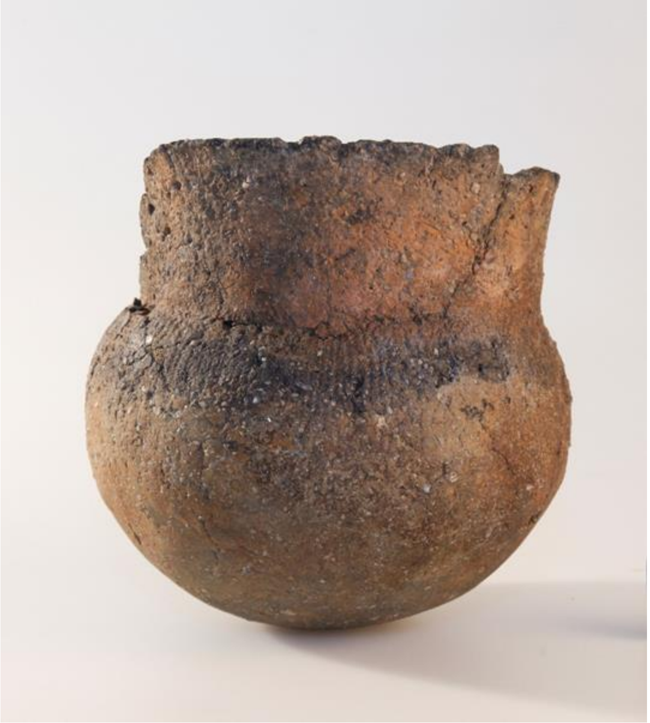

Fig SI.13.1. A ceramic funnel beaker, 17 cm high and 16 cm wide, of Neolithic date (Nordjyllands Kystmuseum number: 29529) fished up from the open sea floor around 40 km north of the contemporary northern tip of Jutland, NW Denmark. Photo: Nordjyllands Kystmuseum.

There is in fact much evidence indicating that such traffic, from Vendsyssel to the coasts of southern Norway and western Sweden, has occurred since the Early Neolithic, as is evident from the discovery of a causewayed enclosure at Hamremoen, near Kristiansand in southernmost Norway. The closest parallel for this kind of monumental structure is found in northern Jutland, where there are also similarities in the ceramic style with that at Hamremoen, which is characterised by decoration consisting of twisted cord impressions in loops in the Volling style [130] (c.f. SI.1). That many such sea crossings were successfully undertaken during the Early and Middle Neolithic is also demonstrated by the numerous flint axes of South Scandinavian origin that ended up in Norway and Sweden. These objects represent an exchange network that extended more than 1000 km, from northern Jutland to northern Norway and Sweden [131–137]. Of particular importance in this context is the distribution of the thin-butted flint axe type, which is coeval with Vittrup Man [17,138] (Figs SI.13.2 and SI.13.3).

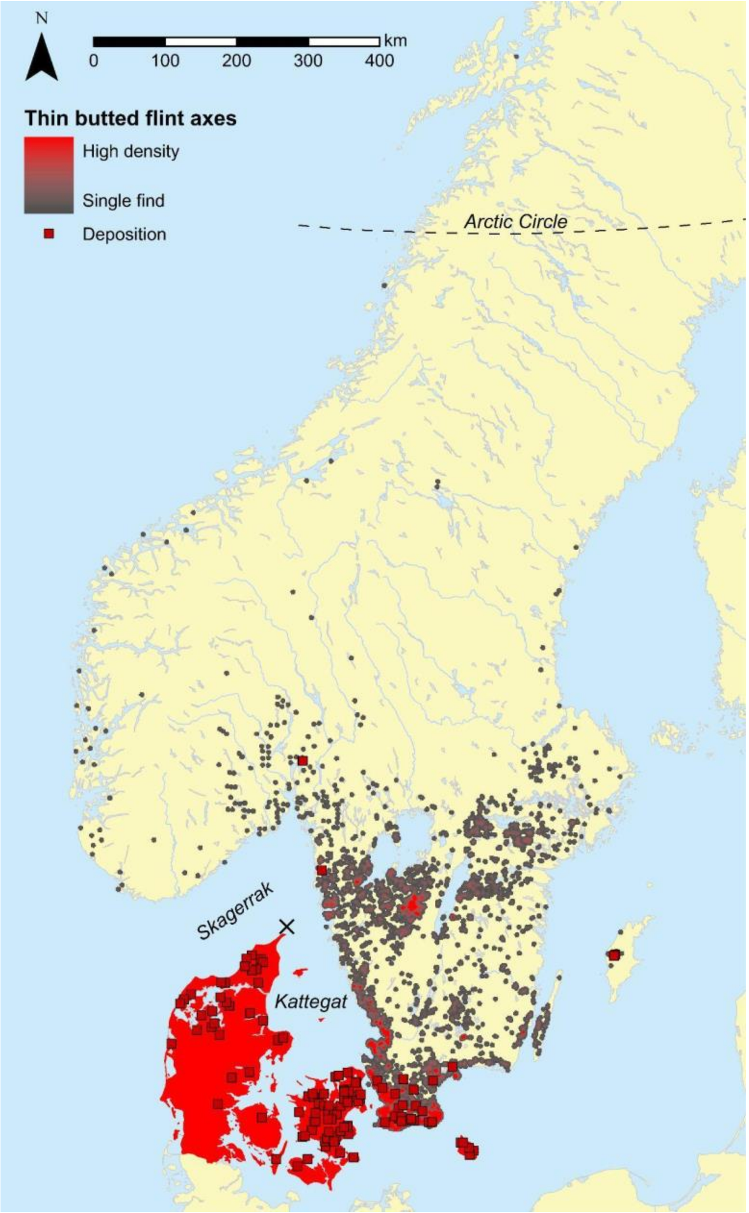

Fig SI.13.2. The distribution of thin-butted axes, including hoard depositions, documenting exchange from the flint-rich regions of South Scandinavia to more northern parts of Scandinavia. The latter areas lack naturally occurring flints of suitable sizes and quality to produce Neolithic-type axes. After: [17,132–135,137,139,140]. The cross marks the findspot where a funnel beaker (Fig SI.13.1) was fished up from the sea at a depth of 120 m. Map data acquired in 2009 from MapCruzin.com.

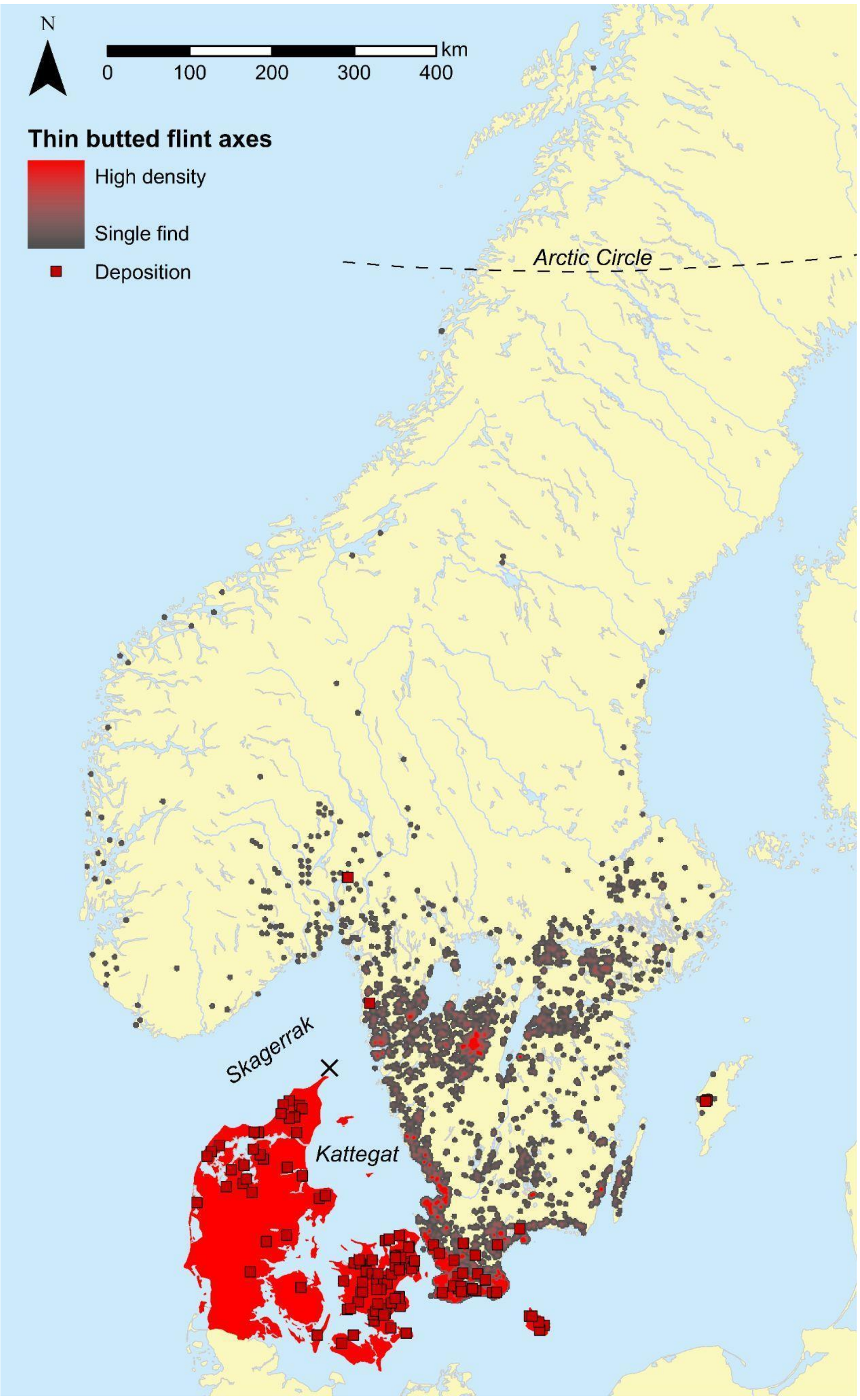

Fig SI.13.2. The distribution of thin-butted axes, including hoard depositions, documenting exchange from the flint-rich regions of South Scandinavia to more northern parts of Scandinavia. The latter areas lack naturally occurring flints of suitable sizes and quality to produce Neolithic-type axes. After: [17,132–135,137,139,140]. The cross marks the findspot where a funnel beaker (Fig SI.13.1) was fished up from the sea at a depth of 120 m. Map data acquired in 2009 from MapCruzin.com.

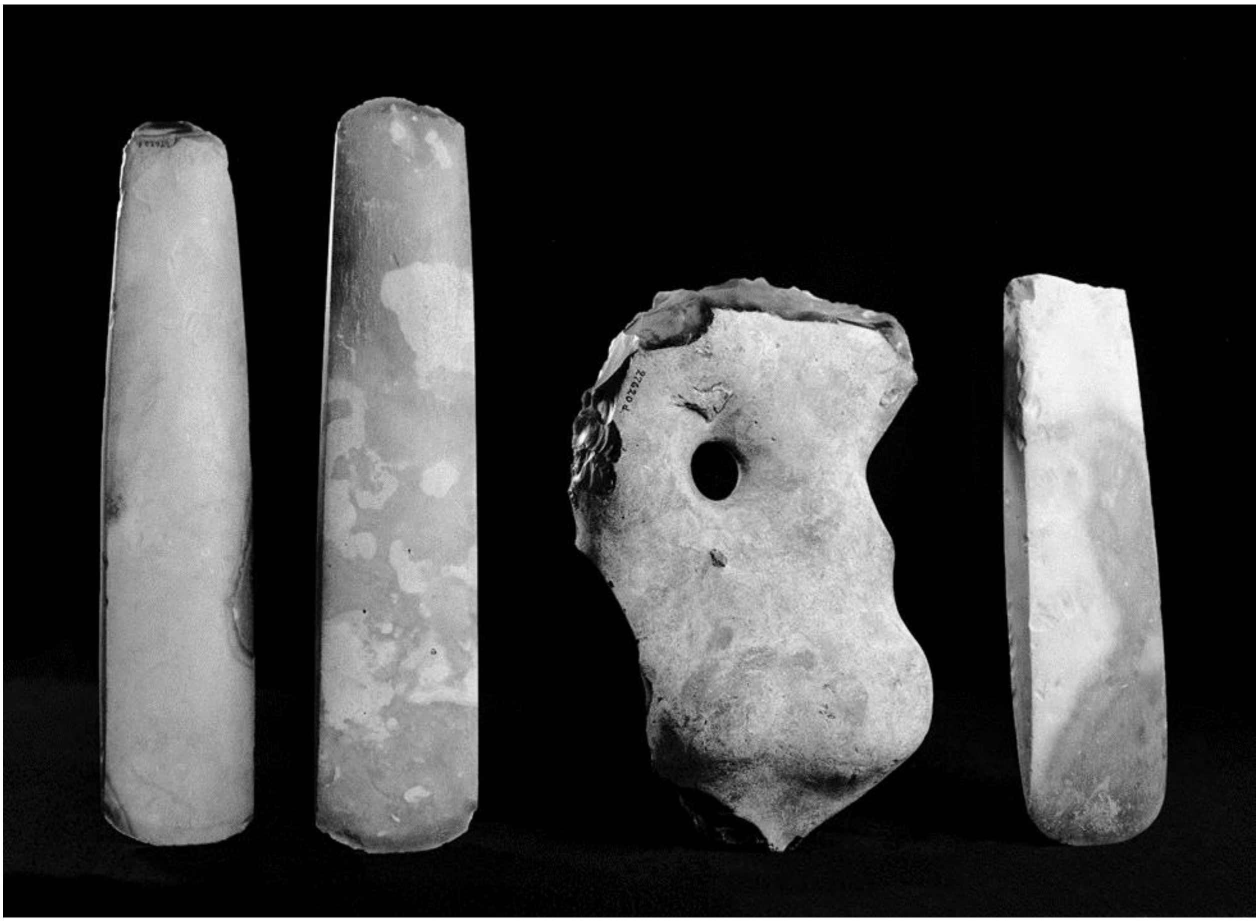

Fig SI.13.3. An example of the kind of material that was traded northwards from the flint-rich parts of Southern Scandinavia: an assemblage, consisting of three thin-butted flint axes and a large flint nodule. These were originally deposited in a bog/wetland at Disen in the inner part of the Oslo Fjord region, Norway. The largest axe is 39.7 cm long. The deposition in Disen represents a tradition of hoarding axes in wetland areas, which is a typical Funnel Beaker Culture practice. Photo: Museum of Cultural History in Oslo, UiO; originally after Glørstad [136]; here reproduced from Sørensen [17].

Many artefacts of Norwegian origin have been recorded in northern Jutland. They document the existence of a network that encompassed the western coast of Norway and was especially active during the earliest part of the South Scandinavian Middle Neolithic – the time of Vittrup Man. As early as 1939, P.V. Glob published some unusual looking adzes made of basalt, which based on comparative studies, he interpreted as being of Norwegian origin [141] (Fig SI.13.4). The adzes can be classified as Vestland adzes that come from the south-western part of Norway [142]. They were probably used for activities such as the scooping or hollowing out timbers and may therefore have been important tools for making dugout canoes [135]. The adzes are all made from regionally available basalt sources with different petrographic characteristics, which could reveal the exact quarried outcrops. The actual dating of the Vestland adzes from North Jutland is somewhat problematic, as most are stray finds. The earliest Vestland adzes in Norway have been found in settlement layers containing

charcoal, radiocarbon dated between 3500 and 2800 cal BC [143,144], thus making the layers coeval with the Vittrup individual. The Vestland adzes found in North Jutland could therefore be interpreted as representing a return flow of material within an exchange system in which Vittrup Man was in some way involved (Fig SI.13.5).

Other types of stray finds of Norwegian or Northern Scandinavian origin have also been found in northern Jutland. A barbed and tanged arrowhead made of slate was found near Strandby in the Himmerland region and another example is reported to have been found in the Mols area of northern Jutland (Fig SI.13.6) [141,145]. Such arrowheads were generally used from the Mesolithic and well into the Neolithic [17]. The barbed and tanged arrowhead from Strandby represents a type that is without a ridge and has hanging barbs, which are diagnostic features of these artefacts at the beginning of the Middle Neolithic in western Norway and Sweden, corresponding with the date of Vittrup Man [146,147]. Another object is an arrowhead of quartz, possibly of Northern Scandinavian origin, which was found at Porshede, in Himmerland, northern Jutland [148]. A final type of stray find was also found in Himmerland, northern Jutland: a single-edged, short-bladed knife made of slate (Fig SI.13.7). This type is also common along the western coast of Norway, but generally further north than the Vestland adzes, and – according to the conventional, South Scandinavian archaeological chronology – dates to between the Mesolithic and the Late Neolithic [149] (Fig SI.13.5). To conclude, there is evidence of continuous contacts across the sea between northern Jutland and southern and western parts of Norway at the time when Vittrup Man lived, as well as during previous and subsequent parts of the Neolithic [17,150].

According to direct radiocarbon dates of charred cereals, farming was practised in southern Norway when the individual found at Vittrup was alive. Finds of naked barley and emmer wheat have been dated to the earliest part of the Middle Neolithic (MNA) (*Hordeum vulgare* var. nudum: Ua-52925: 4551±56 BP, 3498–3035 cal BC; *Triticum dicoccum*: Ua-52926: 4351±55 BP, 3310–2800 cal BC) [151–153]. Could the appearance of the earliest farm products in southern Norway have been the result of these seafaring travels and bartering networks? Was the Vittrup individual searching for flint axes and agricultural products in return for exotic basalt adzes and other commodities that are not preserved in the archaeological record? Or was he taken as a slave (SI.14) as part of exchange of goods between the hunter-gatherer groups of the Scandinavian Peninsula and farmers in northern Jutland? These questions remain unanswered. What is clear is that farmers in the Vittrup area of northern Jutland and hunter-gatherer groups in western parts of Norway were in contact and were also involved in reciprocal exchange. The distribution of the thin-butted flint axes, basalt adzes and knives, as well as arrowheads made of slate, reflects a routinisation of sea voyages from the Early Neolithic onwards between northern Jutland and the south-western coast of Norway. From here, it would have been possible to follow the coastlines and sail further inland following the major rivers. The life-history of Vittrup Man can be seen in relation to such Middle Neolithic seafaring.

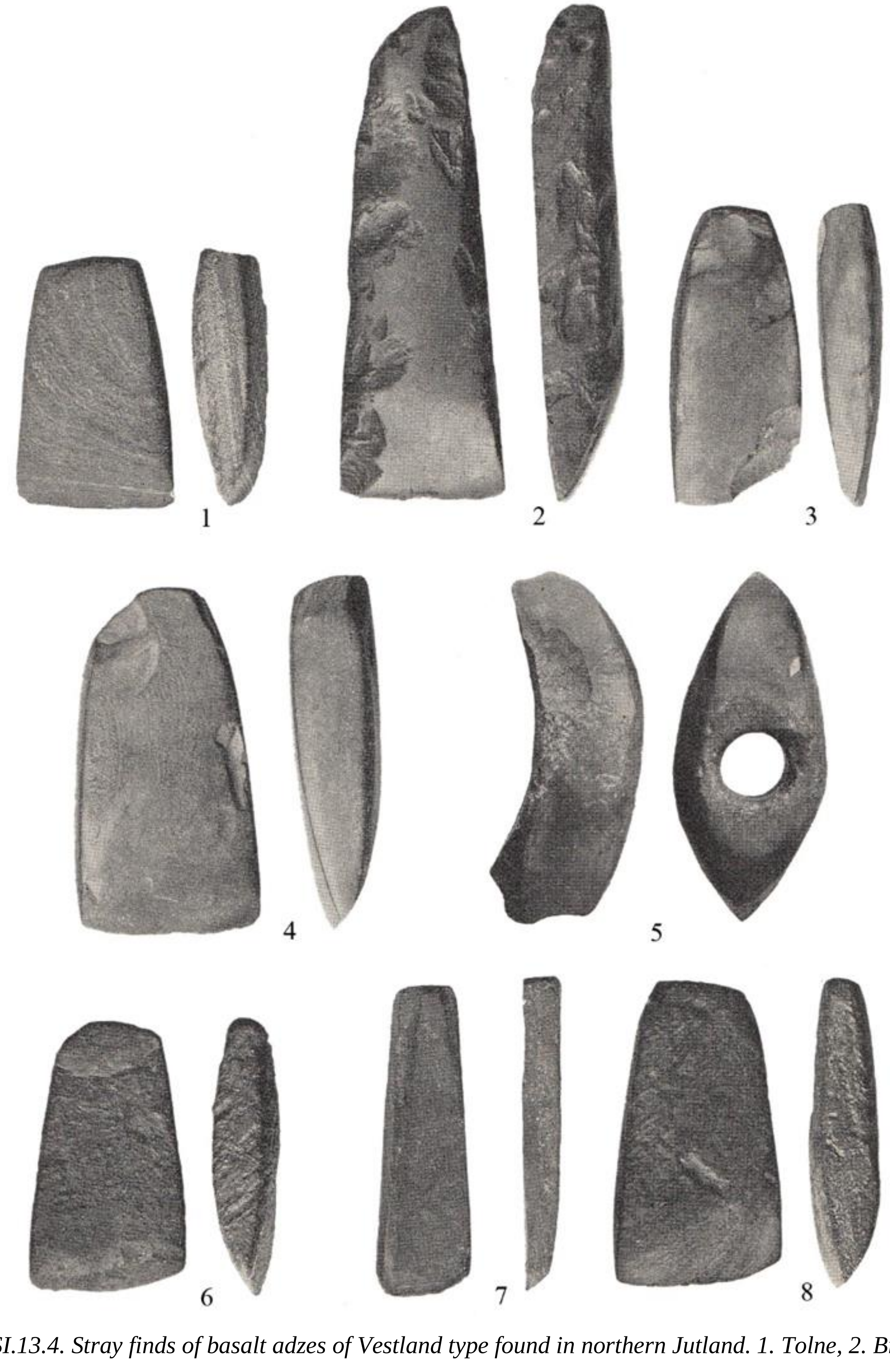

Fig SI.13.4. Stray finds of basalt adzes of Vestland type found in northern Jutland. 1. Tolne, 2. Biersted, 3. Gravlev, 4. Maarup, 5. Raabjerg (battle axe made of basalt), 6. Astrup, 7. Hallund and 8. Sulsted. Photo: The National Museum of Denmark. After [141].

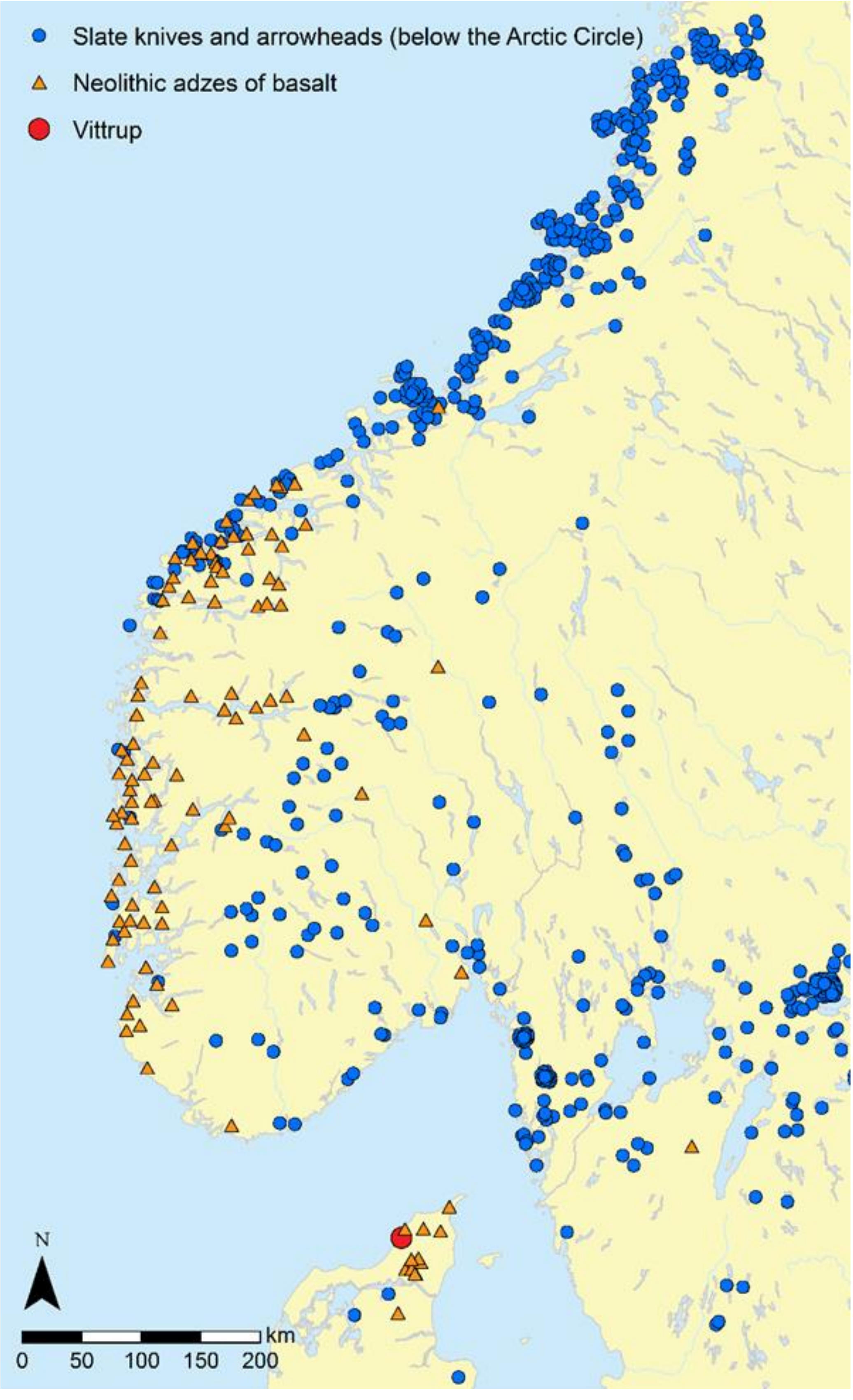

Fig SI.13.5. Distribution of Neolithic knives and arrowheads of slate and adzes of basalt. After: [141,142,146,149,154]. Map data acquired in 2009 from MapCruzin.com.

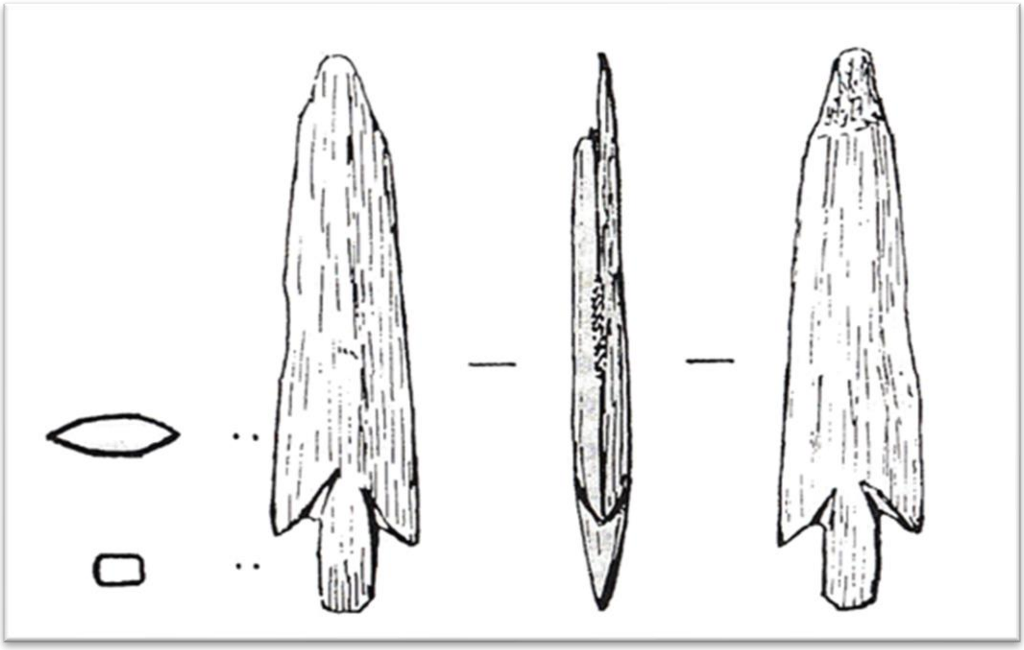

Fig SI.13.6. Stray find of a barbed and tanged arrowhead made of slate from Myrhøj, northern Jutland. After [145]. Drawing: The National Museum of Denmark.

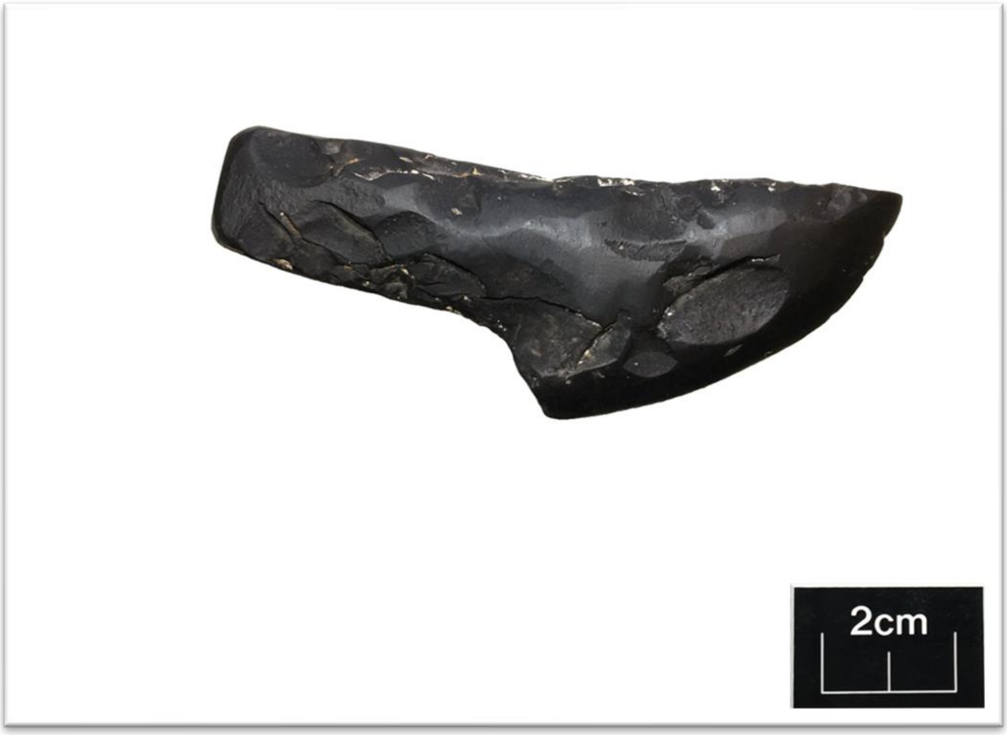

Fig SI.13.7. Stray find of a single-edged, short-bladed knife made of slate found near Nibe, northern Jutland. Photo: Danish National Museum.

SI.14: Vittrup Man a slave?

Anders Fischer and Kristian Kristiansen

Enslavement appears to have been a frequent phenomenon in the global past. However, we need to distinguish between the role of slaves/unfree in pre-state and state societies. In pre-state societies slaves were usually not traded, but rather the results of capture from external raiding, or from losing your freedom internally through debt. A recent comparative study of captives in North and South American pre-state societies [155] has documented that captives amounted to 10–30% of the population.

We suggest that slavery should be considered a central aspect of the neolithization and of the subsequent social dynamics of the European continent. However, we recognize that captives/slaves are notoriously difficult to demonstrate in the archaeological record, unless we have specific conditions of skeletal preservation and circumstantial evidence as in the case of Vittrup Man [156–158]. The taking of captives is usually done from outside your own ethnic group, or from groups that you are not currently allied with [155,159–161]. Thus, we may envisage trading and raiding for captives as two sides of the same coin (e.g. [162]. The situation in Scandinavia during the late 4th millennium BC, when different archaeological/ethnic groups co-existed, provided a classic situation for both peaceful and hostile interactions, leading to raiding for captives. Archaeological indications of cultural interaction, both peaceful and hostile, between Funnel Beaker Culture (farmer) and Pitted Ware Culture (primarily fisher-hunter-gatherer) groups during centuries with little or no genetic admixture are well documented [55,56,84,163]. We suggest that it reflects a complicated and contentious relationship between these groups, that included captives and slaves more often than the rare skeletal evidence bear witness to.

The inference of Vittrup Man as a slave and the potentially widespread existence of slavery in the Scandinavian Neolithic should not be highly controversial. At least, the practice of bondage appears to be demonstrated in Central European multi-person burials from Neolithic societies that were cultural predecessors of the Funnel Beaker Culture [17,164–166].

References

1. Andersen STH. The bog find from Sigersdal. Comments from the excavator. Journal of Danish Archaeology. 1987;6: 220–222.

2. Brøndsted J. Danmarks oldtid: Stenalderen. Gyldendal; 1938.

3. Becker CJ. Mosefundne lerkar fra yngre stenalder: studier over tragtbægerkulturen i Danmark. Aarbøger for Nordisk Oldkyndighed og Historie. 1947; 1–318.

4. Bidstrup K. Holger Friis fortæller om et muntert og virksomt liv som museumsmand, arkæolog og tandlæge. ff., København; 1975.

5. Friis H, Lysdahl P. Har der virkelig fundet menneskeofringer sted i oldtidens Vendsyssel? Et radioforedrag fra 1940. Vendsyssel nu og da 1998-1999. 1999; 4–15.

6. Andersen S, Sjørring S. Geologisk set: Det nordlige Jylland: En beskrivelse af områder af national geologisk interesse. Brenderup: Geografforlaget/Miljøministeriet, Skov-og Naturstyrelsen; 1992.

7. Richardt N. Sedimentological examination of the Late Weichselian sea-level history following deglaciation of northern Denmark. In: Andrews JT, N AWE, Bergsten H, Jennings AE, editors. Late Quaternary Palaeoceanography of the North Atlantic Margins. Geological Society London Special Publications No. 111; 1996. pp. 261–273.

8. Jessen A. Beskrivelse til Geologisk Kort over Danmark (i Maalestok 1:100,000). Kortbladene Skagen, Hirshals, Frederikshavn, Hjørring og Løkken. Danmarks geologiske Undersøgelse. I. Række; 1899.

9. Penney DN. The Holocene marine sequence in the Løkken area of Vendsyssel, Denmark. Eiszeitalter und Gegenwart. 1985;35: 79–88.

10. Aaby B. Geologi og mosedannelse i Store Vildmose området. In: Barfod L, Brix B, Hansen P, editors. Landet og loven Tilegnet Viggo Nielsen på 70-årsdagen 25 januar 1990. 1990. pp. 145–151.

11. Fischer A, Petersen PV. Denmark – a sea of archaeological plenty. In: Fischer A, Pedersen L, editors. Oceans of Archaeology. Jutland Archaeological Society; 2018. pp. 68–83.

12. Mertz EL. De sen- og postglaciale Niveauforandringer i Danmark. Danmarks geologiske Undersøgelser. 1924;41.

13. Christensen C, Nielsen AB. Dating Littorina sea shore levels in Denmark on the basis of data from a Mesolithic coastal settlement on Skagens Odde, northern Jutland. Polish Geological Institute Special Papers. 2008;23: 27–38.

14. Mathiassen T. Nordvestsjællands Oldtidsbebyggelse. Nationalmuseets Skrifter Arkæologisk-Historisk Række VII. 1959.

15. Andersen ST, Rasmussen KL. Radiocarbon wiggle-dating of elm declines in northwest Denmark and their significance. Veg Hist Archaeobot. 1993;2: 125–135.

16. Jørgensen E. Yngre stenalder. In: Ethelberg, P., Jørgensen, E., Meier, D. and Robinson, D, editor. Det Sønderjyske Landbrugs Historie Sten- og Bronzealder. Haderslev: Haderslev Museum and Historisk Samfund for Sønderjylland; 2000. pp. 63–133.

17. Sørensen L. From hunter to farmer in Northern Europe: Migration and adaptation during the Neolithic and Bronze Age, vol. 1+2. Randsborg K, Kaul F, editors. Ph.D, Wiley. 2014.

18. Lysdahl P. Oldtiden i Hirtshals Kommune. En kortfattet oversigt. Lokalhistorisk Årbog Fra Egnens Fortid. 1985; 5–36.

19. Ebbesen K. Tragtbægerkultur i Nordjylland: studier over jættestuetiden. Det Kongelige Nordiske Oldskriftselskab; 1978.

20. Ebbesen K. Danmarks Megalitgrave: Bind 2. Katalogue. Attika; 2008.

21. Sjögren K-G, Fischer A. The chronology of Danish dolmens. Results from 14C dates on human bones. Journal of Neolithic Archaeology.

22. Madsen T, Petersen JE. Tidligneolitiske anlæg ved Mosegården. Regionale og kronologiske forskelle i tidligneolitikum. Kuml. 1983;31: 61–120.

23. Kristensen IK. Storgard IV: An Early Neolithic Long Barrow near Fjelsø, North Jutland. J Dan Archaeol. 1989;8: 72–87.

24. Ebbesen K. Simple, tidligneolitiske grave. Aarbøger for nordisk Oldkyndighed og Historie. 1994; 47–102.

25. Skousen H. Arkæologi i lange baner: undersøgelser forud for anlæggelsen af motorvejen nord om Århus 1998-2007. Moesgård; 2008.

26. Ravn M. The Early Neolithic Volling site of Kildevang—its chronology and intra-spatial organisation. Frühe Keramik im Ostseeraum—Datierung und Sozialer Kontext Internationaler Workshop in Schleswig vom. 2011. pp. 135–163.

27. Nilssen T. En stormandsgrav fra Brønderslevs stenalder. Vendsyssel nu & da 1996. 1996.

28. Rasmussen KL. Danske arkæologiske 14C-dateringer, København 1996. Arkæologiske udgravninger i Danmark 1996. 1997; 286–299.

29. Battinger C. Vedanatomisk analyse af trækølle fra Vittrup Mose. NNU rapport 18. National Museum of Denmark, Copenhagen/Brede; 2019.

30. Munk H. Hasselskoven. En skov- og landbrugskulturhistorisk studie fra Sydsjælland i 1660-1700. Copenhagen: Holger Munk; 1969.

31. Brøndegaard VJ. Folk og flora: Bind 2. Rosenkilde og Bagger, Copenhagen; 1979.

32. Sand-Jensen K, Friis Møller P. Naturen i Danmark 4. Skovene. Gyldendal; 2010.

33. Fischer A, Mortensen MF, Henriksen PS, Mathiassen DR, Olsen J. Dating the Trollesgave site and the Bromme culture – chronological fix-points for the Lateglacial settlement of Southern Scandinavia. J Archaeol Sci. 2013;40: 4663–4674.

34. Aaris-Sørensen K. Diversity and dynamics of the mammalian fauna in Denmark throughout the last glacial-interglacial cycle, 115-0 kyr BP. John Wiley & Sons; 2010.

35. Fischer A, Olsen J, Richards M, Heinemeier J, Sveinbjornsdottir A, Bennike P. Coast–inland mobility and diet in the Danish Mesolithic and Neolithic: evidence from stable isotope values of humans and dogs. J Archaeol Sci. 2007;34: 2125–2150.

36. Mortensen MF, Christensen C, Johannesen K, Stidsing E, Fiedel R, Olsen J. Iron Age peat cutting and ritual depositions in bogs – new evidence from Fuglsøgaard Mose, Denmark. Dan J Archaeol. 2020;9: 1–30.

37. Schour I, Massler M. Studies in Tooth Development: The Growth Pattern of Human Teeth Part II. The Journal of the American Dental Association. 1940;27: 1918–1931.

38. Haavikko K. The formation and the alveolar and clinical eruption of the permanent teeth. An orthopantomographic study. Suom Hammaslaak Toim. 1970;66: 103–170.

39. Harris EF, Buck AL. Tooth Mineralization: A Technical Note on the Moorrees-Fanning-Hunt Standards. DAJ. 2002;16: 15–20.

40. Smith BH. Patterns of molar wear in hunger-gatherers and agriculturalists. Am J Phys Anthropol. 1984;63: 39–56.

41. Alexandersen V. The late Mesolithic dentition in southern Scandinavia. Rivista di Anthropologia. 1988; 191–204.

42. Tribble GD, Kerr JE, Wang B-Y. Genetic diversity in the oral pathogen Porphyromonas gingivalis: molecular mechanisms and biological consequences. Future Microbiol. 2013;8: 607–620.

43. Jersie-Christensen RR, Lanigan LT, Lyon D, Mackie M, Belstrøm D, Kelstrup CD, et al. Quantitative metaproteomics of medieval dental calculus reveals individual oral health status. Nat Commun. 2018;9: 4744.

44. Tyanova S, Temu T, Carlson A, Sinitcyn P, Mann M, Cox J. Visualization of LC-MS/MS proteomics data in MaxQuant. Proteomics. 2015;15: 1453–1456.

45. Warinner C, Rodrigues JFM, Vyas R, Trachsel C, Shved N, Grossmann J, et al. Pathogens and host immunity in the ancient human oral cavity. Nat Genet. 2014;46: 336–344.

46. Bleasdale M, Richter KK, Janzen A, Brown S, Scott A, Zech J, et al. Ancient proteins provide evidence of dairy consumption in eastern Africa. Nat Commun. 2021;12: 632.

47. Scorrano G, Nielsen SH, Lo Vetro D, Mackie M, Margaryan A, Fotakis AK, et al. Genomic ancestry, diet and microbiomes of Upper Palaeolithic hunter-gatherers from San Teodoro cave (Sicily, Italy). bioRxiv. 2021. p. 2021.12.08.471745. doi:10.1101/2021.12.08.471745

48. Altschul SF, Gish W, Miller W, Myers EW, Lipman DJ. Basic local alignment search tool. J Mol Biol. 1990;215: 403–410.

49. Mackie M, Rüther P, Samodova D, Di Gianvincenzo F, Granzotto C, Lyon D, et al. Palaeoproteomic Profiling of Conservation Layers on a 14th Century Italian Wall Painting. Angew Chem Int Ed Engl. 2018;57: 7369–7374.

50. Cappellini E, Welker F, Pandolfi L, Ramos-Madrigal J, Samodova D, Rüther PL, et al. Early Pleistocene enamel proteome from Dmanisi resolves Stephanorhinus phylogeny. Nature. 2019;574: 103–107.

51. Welker F, Ramos-Madrigal J, Gutenbrunner P, Mackie M, Tiwary S, Rakownikow Jersie-Christensen R, et al. The dental proteome of Homo antecessor. Nature. 2020;580: 235–238.

52. Perez-Riverol Y, Bai J, Bandla C, García-Seisdedos D, Hewapathirana S, Kamatchinathan S, et al. The PRIDE database resources in 2022: a hub for mass spectrometry-based proteomics evidences. Nucleic Acids Res. 2022;50: D543–D552.

53. Degerbøl M, Fredskild B. The Urus (*Bos primigenius* Bojanus) and neolithic domesticated cattle (*Bos taurus domesticus* Linné) in Denmark: With a revision of *Bos*-remains from the kitchen middens; Zoological and palynological investigations. Munksgaard; 1970.

54. Johannsen NN. Palaeopathology and Neolithic cattle traction: methodological issues and archaeological perspectives. In: Davies J, Fabiš M, Mainland I, Richards M, Thomas R, editors. Diet and health in past animal populations Current research and future directions, 9th ICAZ Conference, Durham 2002. Oxbow Books, Antony Rowe Ltd. Chippenham; 2005. pp. 39–51.

55. Allentoft ME, Sikora M, Refoyo-Martínez A, Irving-Pease EK, Fischer A, Barrie W, et al. Population genomics of post-glacial western Eurasia. Nature 2024;625: 301–311. https://doi.org/10.1038/s41586-023-06865-0 [Allentoft ME, Sikora M, Refoyo-Martínez A, Irving-Pease EK, Fischer A, Barrie W, et al. Population genomics of post-glacial western Eurasia. Nature 2024;625: 301–311. https://doi.org/10.1038/s41586-023-06865-0].

56. Coutinho A, Günther T, Munters AR, Svensson EM, Götherström A, Storå J, et al. The Neolithic Pitted Ware culture foragers were culturally but not genetically influenced by the Battle Axe culture herders. Am J Phys Anthropol. 2020;172: 638–649.

57. Skoglund P, Malmström H, Omrak A, Raghavan M, Valdiosera C, Günther T, et al. Genomic Diversity and Admixture Differs for Stone-Age Scandinavian Foragers and Farmers. Science. 2014;344: 747–750.

58. Malmström H, Günther T, Svensson EM, Juras A, Fraser M, Munters AR, et al. The genomic ancestry of the Scandinavian Battle Axe Culture people and their relation to the broader Corded Ware horizon. Proc Biol Sci. 2019;286: 20191528.

59. 1000 Genomes Project Consortium, Auton A, Brooks LD, Durbin RM, Garrison EP, Kang HM, et al. A global reference for human genetic variation. Nature. 2015;526: 68–74.

60. Li H. A statistical framework for SNP calling, mutation discovery, association mapping and population genetical parameter estimation from sequencing data. Bioinformatics. 2011;27: 2987–2993.

61. Patterson N, Price AL, Reich D. Population structure and eigenanalysis. PLOS Genet. 2006;2: e190.

62. Price AL, Patterson NJ, Plenge RM, Weinblatt ME, Shadick NA, Reich D. Principal components analysis corrects for stratification in genome-wide association studies. Nat Genet. 2006;38: 904–909.

63. Pritchard JK, Stephens M, Donnelly P. Inference of population structure using multilocus genotype data. Genetics. 2000;155: 945–959.

64. Patterson N, Moorjani P, Luo Y, Mallick S, Rohland N, Zhan Y, et al. Ancient admixture in human history. Genetics. 2012;192: 1065–1093.

65. Maier R, Flegontov P, Flegontova O, Changmai P, Reich D. On the limits of fitting complex models of population history to genetic data. bioRxiv. 2022. p. 2022.05.08.491072. doi:10.1101/2022.05.08.491072

66. Purcell S, Neale B, Todd-Brown K, Thomas L, Ferreira MAR, Bender D, et al. PLINK: a tool set for whole-genome association and population-based linkage analyses. Am J Hum Genet. 2007;81: 559–575.

67. Lazaridis I, Patterson N, Mittnik A, Renaud G, Mallick S, Kirsanow K, et al. Ancient human genomes suggest three ancestral populations for present-day Europeans. Nature. 2014;513: 409–413.

68. Günther T, Malmström H, Svensson EM, Omrak A, Sánchez-Quinto F, Kılınç GM, et al. Population genomics of Mesolithic Scandinavia: Investigating early postglacial migration routes and high-latitude adaptation. PLOS Biol. 2018;16: 1–22.

69. Irving-Pease EK, Refoyo-Martínez A, Ingason A. The Selection Landscape and Genetic Legacy of Ancient Eurasians. bioRxiv. 2022. Available: https://www.biorxiv.org/content/10.1101/2022.09.22.509027.abstract [Irving-Pease EK, Refoyo-Martínez A, Barrie W, Ingason A, Pearson A, Fischer A. et al. The selection landscape and genetic legacy of ancient Eurasians. Nature. 2024;625: 312–320. https://doi.org/10.1038/s41586-023-06705-1].

70. Torkamani A, Wineinger NE, Topol EJ. The personal and clinical utility of polygenic risk scores. Nat Rev Genet. 2018;19: 581–590.

71. Rosenberg NA, Edge MD, Pritchard JK, Feldman MW. Interpreting polygenic scores, polygenic adaptation, and human phenotypic differences. Evolution, Medicine, and Public Health. 2019;2019: 26–34.

72. Bycroft C, Freeman C, Petkova D, Band G, Elliott LT, Sharp K, et al. The UK Biobank resource with deep phenotyping and genomic data. Nature. 2018;562: 203–209.

73. Berg JJ, Harpak A, Sinnott-Armstrong N, Joergensen AM, Mostafavi H, Field Y, et al. Reduced signal for polygenic adaptation of height in UK Biobank. Elife. 2019;8. doi:10.7554/eLife.39725

74. Sohail M, Maier RM, Ganna A, Bloemendal A, Martin AR, Turchin MC, et al. Polygenic adaptation on height is overestimated due to uncorrected stratification in genome-wide association studies. Elife. 2019;8. doi:10.7554/eLife.39702

75. Durvasula A, Lohmueller KE. Negative selection on complex traits limits phenotype prediction accuracy between populations. Am J Hum Genet. 2021;108: 620–631.

76. Refoyo-Martínez A, Liu S, Jørgensen AM, Jin X, Albrechtsen A, Martin AR, et al. How robust are cross-population signatures of polygenic adaptation in humans? Peer Community Journal. 2021;1: 1–None.

77. Allentoft ME, Sikora M, Sjögren K-G, Rasmussen S, Rasmussen M, Stenderup J, et al. Population genomics of Bronze Age Eurasia. Nature. 2015;522: 167–172.

78. Jensen TZT, Niemann J, Iversen KH, Fotakis AK, Gopalakrishnan S, Vågene ÅJ, et al. A 5700 year-old human genome and oral microbiome from chewed birch pitch. Nat Commun. 2019;10: 5520.

79. Margaryan A, Lawson DJ, Sikora M, Racimo F, Rasmussen S, Moltke I, et al. Population genomics of the Viking world. Nature. 2020;585: 390–396.

80. Egfjord AF-H, Margaryan A, Fischer A, Sjögren K-G, Price TD, Johannsen NN, et al. Genomic Steppe ancestry in skeletons from the Neolithic Single Grave Culture in Denmark. PLOS One. 2021;16: e0244872.

81. Pearson K. Principal components analysis. The London, Edinburgh, and Dublin Philosophical Magazine and Journal of Science. 1901;6: 559.

82. Wilhelmson H, Ahlström T. Iron Age migration on the island of Öland: Apportionment of strontium by means of Bayesian mixing analysis. J Archaeol Sci. 2015;64: 30–45.

83. Blank M, Sjögren K-G, Knipper C, Frei KM, Storå J. Isotope values of the bioavailable strontium in inland southwestern Sweden—A baseline for mobility studies. PLOS One. 2018;13: e0204649.

84. Klassen L. The Pitted Ware Culture on Djursland: Supra-regional Significance and Contacts in the Middle Neolithic of Southern Scandinavia. Aarhus University Press; 2020.

85. Ladegaard-Pedersen P, Sabatini S, Frei R, Kristiansen K, Frei KM. Testing Late Bronze Age mobility in southern Sweden in the light of a new multi-proxy strontium isotope baseline of Scania. PLOS One. 2021;16: e0250279.

86. Ahlström T, Price TD. Mobile or stationary? An analysis of strontium and carbon isotopes from Västerbjers, Gotland, Sweden. Journal of Archaeological Science: Reports. 2021;36: 102902.

87. Harrison RG, Katzenberg MA. Paleodiet studies using stable carbon isotopes from bone apatite and collagen: examples from Southern Ontario and San Nicolas Island, California. Journal of Anthropological Archaeology. 2003;22: 227–244.

88. Tykot RH, Falabella F, Planella MT, Aspillaga E, Sanhueza L, Becker C. Stable isotopes and archaeology in central Chile: methodological insights and interpretative problems for dietary reconstruction. Int J Osteoarchaeol. 2009;19: 156–170.

89. Ambrose SH, Norr L. Experimental Evidence for the Relationship of the Carbon Isotope Ratios of Whole Diet and Dietary Protein to Those of Bone Collagen and Carbonate. In: Lambert JB, Grupe G, editors. Prehistoric Human Bone: Archaeology at the Molecular Level. Berlin, Heidelberg: Springer Berlin Heidelberg; 1993. pp. 1–37.

90. Passey BH, Robinson TF, Ayliffe LK, Cerling TE, Sponheimer M, Dearing MD, et al. Carbon isotope fractionation between diet, breath CO2, and bioapatite in different mammals. J Archaeol Sci. 2005;32: 1459–1470.

91. Sjögren KG, Price TD. Vegetarians or meat eaters? Enamel δ13C and Neolithic diet at the Frälsegården passage tomb, central Sweden. In: Bergerbrant S, Sabatini S, editors. Counterpoint: Essays in Archaeology and Heritage Studies in Honour of Professor Kristian Kristiansen. Oxford: Archaeopress; 2013. pp. 690–704.

92. DeNiro MJ. Postmortem preservation and alteration of in vivo bone collagen isotope ratios in relation to palaeodietary reconstruction. Nature. 1985;317: 806–809.

93. Ambrose SH, DeNiro MJ. The isotopic ecology of East African mammals. Oecologia. 1986;69: 395–406.

94. Brown TA, Nelson DE, Vogel JS, Southon JR. Improved Collagen Extraction by Modified Longin Method. Radiocarbon. 1988;30: 171–177.

95. Reimer P, Hoper S, McDonald J, Reimer R, Svyatko S, Thompson M. Laboratory Protocols used for AMS Radiocarbon Dating at the 14Chrono Centre. Research Report Series, The Queen’s University, Belfast; 2015.

96. van Klinken GJ. Bone Collagen Quality Indicators for Palaeodietary and Radiocarbon Measurements. Journal of Archaeological Science. 1999. pp. 687–695. doi:10.1006/jasc.1998.0385

97. Ramsey CB, Higham T, Bowles A, Hedges R. Improvements to the Pretreatment of Bone at Oxford. Radiocarbon. 2004;46: 155–163.

98. Hedges REM. Isotopes and red herrings: comments on Milner et al. and Lidén et al. Antiquity. 2004;78: 34–37.

99. Eriksson G. Part-time farmers or hard-core sealers? Västerbjers studied by means of stable isotope analysis. Journal of Anthropological Archaeology. 2004;23: 135–162.

100. Lidén K, Eriksson G, Nordqvist B. The wet and the wild followed by the dry and the tame-or did they occur at the same time?: Diet in Mesolithic Neolithic southern Sweden. Antiquity. 2004. Available: http://swepub.kb.se/bib/swepub:oai:DiVA.org:uu-72619

101. Fornander E, Eriksson G, Lidén K. Wild at heart: Approaching Pitted Ware identity, economy and cosmology through stable isotopes in skeletal material from the Neolithic site Korsnäs in Eastern Central Sweden. Journal of Anthropological Archaeology. 2008;27: 281–297.

102. Eriksson G, Frei KM, Howcroft R, Gummesson S, Molin F, Lidén K, et al. Diet and mobility among Mesolithic hunter-gatherers in Motala (Sweden) - The isotope perspective. Journal of Archaeological Science: Reports. 2018;17: 904–918.

103. Robson HK, Andersen SH, Clarke L, Craig OE, Gron KJ, Jones AKG, et al. Carbon and nitrogen stable isotope values in freshwater, brackish and marine fish bone collagen from Mesolithic and Neolithic sites in central and northern Europe. Environ Archaeol. 2016;21: 105–118.

104. Gron KJ, Rowley-Conwy P. Herbivore diets and the anthropogenic environment of early farming in southern Scandinavia. Holocene. 2017;27: 98–109.

105. Sjögren K-G. Modeling middle Neolithic funnel beaker diet on Falbygden, Sweden. Journal of Archaeological Science: Reports. 2017;12: 295–306.

106. Gron KJ, Larsson M, Gröcke DR, Andersen NH, Andreasen MH, Bech J-H, et al. Archaeological cereals as an isotope record of long-term soil health and anthropogenic amendment in southern Scandinavia. Quat Sci Rev. 2021;253: 106762.

107. Fischer A, Gotfredsen AB, Meadows J, Pedersen L, Stafford M. The Rødhals kitchen midden – marine adaptations at the end of the Mesolithic world. Journal of Archaeological Science: Reports. 2021;39: 103102.

108. Bogaard A, Heaton THE, Poulton P, Merbach I. The impact of manuring on nitrogen isotope ratios in cereals: archaeological implications for reconstruction of diet and crop management practices. J Archaeol Sci. 2007;34: 335–343.

109. Kanstrup M, Holst MK, Jensen PM, Thomsen IK, Christensen BT. Searching for long-term trends in prehistoric manuring practice. $\delta 15N$ analyses of charred cereal grains from the 4th to the 1st millennium BC. J Archaeol Sci. 2014;51: 115–125.

110. Gron KJ, Montgomery J, Rowley-Conwy P. Cattle management for dairying in Scandinavia's earliest Neolithic. PLoS One. 2015;10: e0131267.

111. Robson HK, Saul H, Steele VJ, Meadows J, Otto Nielsen P, Fischer A, et al. Organic residue analysis of Early Neolithic "bog pots" from Denmark demonstrates the processing of wild and domestic foodstuffs. J Archaeol Sci Rep. 2021;36: 102829.

112. Pedersen L. Eelers in Danish waters–interaction between men and their marine environment over 8000 years. In: Daire M-Y, Dupont C, Baudry A, Billard C, Large J-M, Lespez L, et al., editors. Ancient maritime communities and the relationship between people and environment along the European Atlantic coasts. BAR International Series; 2013. pp. 163–173.

113. Craig OE, Steele VJ, Fischer A, Hartz S, Andersen SH, Donohoe P, et al. Ancient lipids reveal continuity in culinary practices across the transition to agriculture in Northern Europe. Proc Natl Acad Sci U S A. 2011;108: 17910–17915.

114. Montgomery J, Beaumont J, Jay M, Keefe K, Gledhill AR, Cook GT, et al. Strategic and sporadic marine consumption at the onset of the Neolithic: increasing temporal resolution in the isotope evidence. Antiquity. 2013;87: 1060–1072.

115. Beaumont J, A. Gledhill J, Lee-Thorp J, Montgomery J. Childhood diet: a closer examination of the evidence from dental tissues using stable isotope analysis of incremental human dentine. Archaeometry. 2013;55: 277–295.

116. Gron KJ, Rowley-Conwy P, Fernandez-Dominguez E, Gröcke DR, Montgomery J, Nowell GM, et al. A meeting in the Forest: Hunters and farmers at the Coneybury "Anomaly", Wiltshire. Proc Prehist Soc. 2018;84: 111–144.

117. Nehlich O. The application of sulphur isotope analyses in archaeological research: A review. Earth-Sci Rev. 2015;142: 1–17.

118. Nehlich O, Richards MP. Establishing collagen quality criteria for sulphur isotope analysis of archaeological bone collagen. Archaeol Anthropol Sci. 2009;1: 59–75.

119. Bennike O, Jakobsen PR, Hansen JW. Late Quaternary history of Lammefjorden, north-west Sjælland, Denmark. GEUS Bulletin. 2020 [cited 25 Aug 2023]. doi:10.34194/geusb.v44.4630

120. Veizer J. Strontium isotopes in seawater through time. Annu Rev Earth Planet Sci. 1989;17: 141.

121. McArthur JM, Howarth RJ, Bailey TR. Strontium Isotope Stratigraphy: LOWESS Version 3: Best Fit to the Marine Sr-Isotope Curve for 0–509 Ma and Accompanying Look-up Table for Deriving Numerical Age. J Geol. 2001;109: 155–170.

122. Fischer A, Olsen J. The Nekselø fish weir and marine reservoir effect in neolithization period Denmark. Radiocarbon. 2021;63: 805–820.

123. Philippsen B, Iversen R, Klassen L, Klassen L. The Pitted Ware culture chronology on Djursland: New evidence from Kainsbakke and other sites. The Pitted Ware Culture on Djursland. 2020.

124. Iversen R, Philippsen B, Persson P. Reconsidering the Pitted Ware chronology. A temporal fixation of the Scandinavian Neolithic hunters, fishers and gatherers. Praehistorische Zeitschrift. 2021;96: 44–88.

125. Klassen L, Rasmussen U, Kveiborg J, Richards M, Orlando L, Svenning J-C, et al. Ginnerup Revisited. New Excavations at a Key Neolithic Site on Djursland, Denmark. Jnanabha. 2023; 35–65.

126. Bennike P, Ebbesen K, Jørgensen LB, Rowley-Conwy P. The bog find from Sigersdal: human sacrifice in the Early Neolithic. Journal of Danish Archaeology. 1986;5: 85–115.

127. Fischer A. Sacral and profane – diversity in the early prehistoric submarine record. In: Fischer A, Pedersen L, editors. Oceans of Archaeology. Jutland Archaeological Society, Højbjerg; 2018. pp. 162–173.

128. Nielsen LH, Johannessen PN. Facies architecture and depositional processes of the Holocene-Recent accretionary forced regressive Skagen spit system, Denmark. Sedimentology. 2009;56: 935–968.

129. Østmo E. Over Skagerak i steinalderen. Noen refleksjoner om oppfinnelsen av havgående fartøyer i Norden. Viking. 2005.

130. Glørstad H, Sundström L. Hamremoen–an enclosure for the hunter-gatherers. In: Furholt M, Hinz M, Mischka D, Noble G, Olausson D, editors. Landscapes, Histories and Societies in the Northern European Neolithic. Institut für Ur- und Frühgeschichte der CAU Kiel; 2014.

131. Becker CJ. Die Nordschwedischen Flintdepots. Acta Archaeologica. 1952;23: 31–79.

132. Hinsch E. Traktbegerkultur - megalitkultur: En studie av Øst-Norges eldste, neolitiske gruppe. Universitetets Oldsaksamling Årbok 1951-1953. 1955.

133. Østmo E. New observations on the funnel beaker culture in Norway. Acta Archaeologica. 1986;55: 190–198.

134. Østmo E. The Northern Periphery of the TRB: Graves and Ritual Deposits in Norway. Acta Archaeol. 2007;78: 111–142.

135. Bergsvik KA, Østmo E. The Experienced Axe: Chronology Condition and Context of TRB: Axes in Western Norway. In: Davis V, Edmonds M, editors. Stone Axe Studies III. Oxbow Books, Oxford; 2011. pp. 7–20.

136. Glørstad H. Traktbegerkulturen i Norge--Kysten, jakten og det tidligste jordbruket. In: Kaul F, Sørensen L, editors. Agrarsamfundenes ekspansion i nord. Nordlige Verdener, The National Museum of Denmark, Copenhagen; 2012. pp. 44–56.

137. Valen CR. Neolitisering av Nord-Norge. Hva sier det arkeologiske gjenstandsmaterialet og de naturvitenskapelige undersøkelsene? In: Kaul F, Sørensen L, editors. Agrarsamfundenes ekspansion i nord. Nordlige Verdener. The National Museum of Denmark, Copenhagen; 2012. pp. 152–168.

138. Nielsen PO. Die Flintbeile der frühen Trichterbecherkultur in Dänemark. Acta Archaeol. 1977;48: 61–138.

139. Oldeberg A. Studien über die schwedische Bootaxtkultur. Wahlström & Widstrand; 1952.

140. Blomqvist L. Neolitisk atlas över västra Götaland. Norders bokhandel, Falköping; 1990.

141. Glob PV. Norske skiferfund i danske fund. Aarbøger for Nordisk Oldkyndighed og Historie. 1939; 296–301.

142. Olsen AB, Alsaker S. Greenstone and diabase utilization in the stone age of western Norway: Technological and socio-cultural aspects of axe and adze production and distribution. Norwegian Archaeological Review. 1984;17: 71–103.

143. Olsen AB. Kotedalen-en boplass gjennom 5000 år: Fangstbosetning og tidlig jordbruk i vestnorsk steinalder: nye funn og nye perspektiver. Historisk museum, Universitetet; 1992.

144. Bergsvik KA. Arkeologiske undersøkelser ved Skatestraumen. Bind 1. Arkeologiske avhandlinger og rapporter fra Universitetet i Bergen – 7. 2002.

145. Ebbesen K. Offerfundet fra Suldrup i Himmerland. Fra Himmerland og Kjær Herred. 1981; 91–109.

146. Taffinder J. The allure of the exotic: The social use of non-local raw materials during the Stone Age in Sweden. Aun 25. Department of Archaeology, Uppsala University; 1998.

147. Olsen TB. The phase of transformation in western Norway. In: McCartan SB, Schulting R, Warren G, Woodman P, editors. Mesolithic Horizons vol II. Oxbow Books, Oxford; 2009. pp. 583–588.

148. Becker CJ. An Arctic-Type arrowhead from North Jutland. Acta Archaeol. 1958;29: 157–161.

149. Søborg. HC. Skiferkniver sør for Polarsirkelen i Norge. En analyse av attributter, typer og geografisk fordeling med bakgrunn i det fennoskandinaviske skiferkompleks. Unpublished MA thesis. University of Bergen; 1986.

150. Sarauw T. Early Late Neolithic Dagger Production in Northern Jutland: marginalised production or source of wealth? Bericht der Römisch-Germanischen Kommission. 2007;87: 213–272.

151. Stokke J-SF, Reitan G. Kvastad A2. Lokalitet med funn fra tidlig- og mellemmesolitikum og dyrkningsspor fra mellem- og senneolitikum. In: Sundström L, Reitan G, editors. Kystens steinalder i Aust-Agder: Arkeologiske undersøkelser i forbindelse med ny E18 Tvedestrand-Arendal. Cappelen Damm Akademisk; 2018. pp. 375–407.

152. Nielsen SV. Early farming in Southeastern Norway: New evidence and interpretations. Journal of Neolithic Archaeology. 2021;23: 83–113.

153. Solheim S. timing the emergence and development of arable farming in southeastern norway by using summed probability distribution of radiocarbon dates and a bayesian age model. Radiocarbon. 2021;63: 1503–1524.

154. Olsen TB. Egger av tid og rom. Transformasjonen av steinalderens fangstsamfunn i Vest-Norge. MA-thesis. Department of Archaeology, University of Bergen; 2004.

155. Cameron CM. Captives: How Stolen People Changed the World. U of Nebraska Press; 2016.

156. Andersen H. Hovedstaden i Riget. Nationalmuseets Arbejdsmark 1960. 1960; 13–35.

157. Naumann E, Krzewińska M, Götherström A, Eriksson G. Slaves as burial gifts in Viking Age Norway? Evidence from stable isotope and ancient DNA analyses. J Archaeol Sci. 2014;41: 533–540.

158. Chinnock C, Marshall M. An Unusual Roman Fettered Burial from Great Casterton, Rutland. Britannia. 2021;52: 175–206.

159. Jewitt JR. White Slaves of the Nootka: Narrative of the Adventures and Sufferings of John R. Jewitt While a Captive of the Nootka Indians on Vancouver Island, 1803-05. Heritage House Pub.; 1987.

160. Vayda AP. Maori warfare. Columbia University; 1956.

161. Silverstein M. Chinookans of the lower Columbia. In: Suttles W, editor. Handbook of North American Indians Vol 7, Northwest Coast. Smithsonian Institution, Washington; 1990. pp. 533–546.

162. Keeley LH. War Before Civilization. Oxford University Press, USA; 1996.

163. Becker CJ. Die mittel-neolithischen Kulturen in Südskandinavien. Acta Archaeologica. 1954; 49–150.

164. Testart A, Jeunesse C, Baray L, Boulestin B. Les esclaves des tombes néolithiques. Pour Sci. 2010;396: 74–80.

165. Jeunesse C. Les sépultures en fosses circulaires de l'horizon 4500 - 3500. Contribution à l'étude comparée des systèmes funéraires du Néolithique européen. In: Baray L, Boulestin B, editors. Morts anormaux et Sépultures bizarres Les dépôts humains en fosses circulaires ou en silos du Néolithique à l'âge du Fer. Éditions universitaires de Dijon; 2010. pp. 28–48.

166. Beau A, Rivollat M, Réveillas H, Pemonge M-H, Mendisco F, Thomas Y, et al. Multi-scale ancient DNA analyses confirm the western origin of Michelsberg farmers and document probable practices of human sacrifice. PLOS ONE. 2017;12: e0179742.
