# Supplementary material for: Vittrup Man–The life-history of a genetic foreigner in Neolithic Denmark
Source: PLoS One. 2024 Feb 14;19(2):e0297032. doi: 10.1371/journal.pone.0297032 (PMC10866469; doi:10.1371/journal.pone.0297032)
Supplement: S2 File — (DOCX) [file pone.0297032.s002.docx]

**S2 File. Deposaries/reproducibility**

The Vittrup site is recorded in the national database for archaeology: Fund & Fortidsminder, at The Danish Agency for Culture and Palaces under the number 100102-56.

The records on the Vittrup site are filed under j. numbers 616/1967 and 54/1968 at Vendsyssel Historiske Museum in Hjørring, Denmark. This museum is also curating the physical remains of Vittrup Man under inventory numbers 11600A-C, whereas the three bovine bones found next to him have inventory numbers 11599A, 11599B and 11602A. Any sample remains of bone and teeth are to be found at Globe Institute, Copenhagen University under inventory number SGG_ 2_015921. A calculus sample from Vittrup Man’s tooth +4 is stored at Globe Institute, Copenhagen University under no. CGG_2_107378.

At the Danish National Museum in Copenhagen the pottery vessel and wooden club from Vittrup has the inventory numbers A28426 and A28427 respectively. At the same place, written files on the site are recorded under j. no. 54/1968.

Proteins data are available via ProteomeXchange (https://www.proteomexchange.org) with identifier PXD044743.

Link to 3D model for the destructively sampled wisdom tooth of Vittrup Man (the OBJ and associated texture files) is available via: <https://doi.org/10.5281/zenodo.7802122>.
